# Virome analysis of an ectomycorrhizal fungus *Suillus luteus* revealing potential evolutionary implications

**DOI:** 10.3389/fcimb.2023.1229859

**Published:** 2023-08-17

**Authors:** Hanzhao Liu, Yifei Zhang, Yingying Liu, Junbo Xiao, Zijie Huang, Yunfeng Li, Huaping Li, Pengfei Li

**Affiliations:** Key Laboratory of Microbial Signals and Disease Control, College of Plant Protection, South China Agricultural University, Guangzhou, Guangdong, China

**Keywords:** *Suillus luteus*, mycovirus, ectomycorrhizal fungus, evolution, virus diversity, metatranscriptome

## Abstract

*Suillus luteus* is a widespread edible ectomycorrhizal fungus that holds significant importance in both ecological and economic value. Mycoviruses are ubiquitous infectious agents hosted in different fungi, with some known to exert beneficial or detrimental effects on their hosts. However, mycoviruses hosted in ectomycorrhizal fungi remain poorly studied. To address this gap in knowledge, we employed next-generation sequencing (NGS) to investigate the virome of *S. luteus*. Using BLASTp analysis and phylogenetic tree construction, we identified 33 mycovirus species, with over half of them belonging to the phylum *Lenarviricota*, and 29 of these viruses were novel. These mycoviruses were further grouped into 11 lineages, with the discovery of a new negative-sense single-stranded RNA viral family in the order *Bunyavirales*. In addition, our findings suggest the occurrence of cross-species transmission (CST) between the fungus and ticks, shedding light on potential evolutionary events that have shaped the viral community in different hosts. This study is not only the first study to characterize mycoviruses in *S. luteus* but highlights the enormous diversity of mycoviruses and their implications for virus evolution.

## 1 Introduction

Mycoviruses are viruses that infect and replicate in fungi. They are widespread throughout all major taxa of fungi (Ghabrial et al., 2015) and have exhibited tremendous diversity in their genome organizations and lifestyles (Kondo et al., 2022). According to the International Committee on Taxonomy of Viruses (ICTV) (The ICTV Virus Metadata Resource of 04/25/2023 version), mycoviruses can be classified into 30 families, including 12 families that possess positive-sense (+) single-stranded (ss) RNA genomes, 10 families with double-stranded (ds) RNA genomes, 5 families with negative-sense (-) ssRNA genomes, 2 families with reverse transcribing ssRNA genomes, and one family with ssDNA genomes. Furthermore, new families with members of distinct features are continually being proposed (Kozlakidis et al., 2013; Deakin et al., 2017; Sutela et al., 2020). Despite variation in genome architectures, the gene coding for RNA-dependent RNA polymerase (RdRp), which is essential for replication, is a highly conserved component in most RNA mycovirus genomes (Ghabrial and Suzuki, 2009; Myers and James, 2022). Mycoviruses usually lack an extracellular route for infection. Therefore, they are transmitted horizontally *via* hyphal anastomosis and vertically through the dissemination of spores (Ghabrial et al., 2015). Most infections caused by mycoviruses are asymptomatic (Ghabrial and Suzuki, 2009), however, some mycoviruses can lead to apparent alteration on their host’s lifestyle (Son et al., 2015). One example is the La-France disease that results in severe yield loss of cultivated mushrooms (*Agaricus bisporus*). The disease is caused by Agaricus bisporus virus 1, which is the first mycovirus discovered in history (Hollings, 1962). Another classic example is Cryphonectria hypovirus 1 (CHV1) which confers hypovirulence to its host, the pathogenic fungus *Cryphonectria parasitica*, and has led to the utilization of CHV1 as an approach to control chestnut blight in Europe (Van Alfen et al., 1975; Anagnostakis, 1982). Such discoveries of ecologic and economic importance have sparked us to further research on mycoviruses and contributed to a better understanding of mycovirus diversity and evolution.

*Suillus luteus* (commonly known as slippery jack) is an edible ectomycorrhizal fungus that establishes a symbiotic relationship with the roots of several *Pinus* species (Pildain et al., 2021). It is natively distributed throughout Eurasia and has been introduced to North and South America, South Africa, and Oceania with plantations of exotic conifers (Policelli et al., 2019). The edibility of *S. luteus* has made it a worthy food source worldwide, with significant commercial and economic value (Sitta and Floriani, 2008; Dai et al., 2010; Barroetaveña and Toledo, 2020). Additionally, *S. luteus* plays a critical role in forest sustainability by exerting a fundamental influence on pine invasions. It is one of the pioneering fungi to first form associations with the roots of conifer seedlings (Chu-Chou and Grace, 1988; Menkis et al., 2005) and facilitates tree colonization of new ranges (Hayward et al., 2015; Policelli et al., 2019). Additionally, by establishing ectomycorrhizal symbioses, *Suillus* species can confer resistance to abiotic and biotic stresses on plants, such as tolerance to low pH (Marx and Zak, 1965), heavy metal (Adriaensen et al., 2005; Ruytinx et al., 2013; Liu et al., 2020), and protection from pathogen attack (Ghorbanpour et al., 2018; Gonthier et al., 2019). These benefits shed light on the profound influence of *S. luteus* on sustainable development and environmental protection, and contribute to the “zero hunger” global goal (Pérez-Moreno et al., 2021). The mycorrhizal system is well known for its rich diversity of microorganisms within its associated microbiomes (van der Heijden et al., 2015; Shi et al., 2023), which makes it an intriguing subject of research. The previous study of *S. luteus*-related biodiversity was mainly focused on the bacteria characterization (Bending et al., 2002; Zhou et al., 2022), however, another layer of complexity contributed by mycoviruses infecting *S. luteus* remains unknown. Therefore, further research on mycoviruses associated with *S. luteus* is warranted.

The utilization of NGS has given us an efficient tool to investigate viral diversity, which has led to a revolutionary understanding of the virosphere (Shi et al., 2016; Dolja and Koonin, 2018; Shi et al., 2018). NGS has been implemented to explore the mycovirome in some economically important pathogenic fungi (Mu et al., 2017; Chen et al., 2021a; Ruiz-Padilla et al., 2021; Sutela et al., 2021; He et al., 2022; Ye et al., 2023) and edible mushrooms (Deakin et al., 2017; Guo et al., 2021), while these fungi only represent a very limited fraction of the entire fungi kingdom. Conversely, mycorrhizal fungi inhabit a distinct environment with immense potential for biodiversity in mycoviruses, but have received relatively little attention. To date, only a few virome studies have targeted mycorrhizal fungi; these include several novel mycoviruses discovered in the metagenomes of ectomycorrhizal fungi *Sarcosphaera coronaria* and *Picoa juniperi* (Sahin et al., 2020; Sahin et al., 2021), and numerous mycoviruses detected in arbuscular mycorrhizal fungi *Gigaspora margarita* and *Rhizophagus* spp. (Neupane et al., 2018; Turina et al., 2018). However, no mycoviruses have been reported in the ectomycorrhizal fungi *Suillus* spp. To fill these research gaps, we used NGS approaches to identify the viral population in *S. luteus*. This work is not only the first study of mycoviruses in *S. luteus* but also provided insight into the overall understanding of mycoviral diversity and evolutionary patterns.

## 2 Materials and methods

### 2.1 Fungal strains isolation and species identification

The dried fruiting bodies of *S. luteus* picked from forests of the Greater Khinggan Mountains of Heilongjiang Province were purchased online in China, then sterilized with 75% (v/v) ethanol. The *S. luteus* isolates were recovered and cultured on Pachlewski’s medium (PACH) at 25°C. All fungal isolates were maintained on PACH plates at 4°C during the studies. 148 strains were acquired. Species identification of each strain was confirmed by the sequencing of the internal transcribed spacer (ITS) regions, which was amplified using primers ITS1F and ITS4 (White et al., 1990; Gardes and Bruns, 1993).

### 2.2 Total RNA extraction and purification

In order to facilitate the collection of mycelium mass, *S. luteus* isolates were cultured on a cellophane membrane overlaying the PACH plate for a week. Each fungal strain was taken with 0.5 g fungal mycelium mass for RNA extraction. Total RNA was extracted with the assistance of the Plant RNA Extraction Kit (Promega, Beijing) following manufacturer instructions. A total of 148 RNA samples were obtained and were reserved at -80˚C for subsequent research. Approximately 2 μg of each RNA sample was collected, mixed, and subsequently sent to Shanghai Biotechnology Corporation (China) for RNA sequencing (RNA-seq).

### 2.3 RNA sequencing and sequence analysis

Total RNA samples were subjected to Ribosomal RNA depletion, then used for library preparation using Zymo-Seq RiboFree Total RNA Library Prep Kit (Zymo Research, USA). Sequencing was performed on the Illumina Hiseq 2000/2500 sequencer by Shanghai Biotechnology Corporation (China). The low-quality reads (including reads shorter than 20 bp, low-quality scores (<20), and adapter sequences) were filtered out from the raw reads obtained. The mRNA sequences of *S. luteus* in the raw reads were filtered out by aligning the reads to the reference sequence of the *S. luteus* strain UH-Slu-Lm8-n1 (https://www.ncbi.nlm.nih.gov/datasets/taxonomy/930992/). The clean reads were *de novo* assembled into Primary UniGenes, using the scaffolding contig algorithm of CLC Genomics Workbench software (version: 6.0.4). The resulting Primary UniGenes were then used to construct the first_contig and the second_contig (with stricter splicing parameters) using CAP3 EST. The contigs obtained were subjected to BLASTx analysis on National Center for Biotechnology Information (NCBI; https://www.ncbi.nlm.nih.gov/) Non-Redundant Protein database.

### 2.4 Confirmation of virus-like contigs

According to the obtained contigs, we designed specific primers to confirm the presence of putative mycoviruses in the strains tested (see **Table S2** for the PCR primers used for viral contig detection). Total RNA samples of 148 strains were subjected to DNase I treatment and reverse transcription (with random primers) using EasyScript One-Step gDNA Removal and cDNA Synthesis SuperMix (TransGen Biotech, Beijing, China). The synthesized cDNA was subsequently amplified using 2×GS Taq PCR Mix (Genesand Biotech Co.,Ltd, Beijing, China) with designed specific primers (**Table S2**).

### 2.5 Bioinformatics analysis

Open reading frames (ORFs) were detected and translated in the translation overview program of DNAMAN software (Version 9). Conserved domains of the amino acid (aa) sequence were detected in the conserved domain (CD) search service on NCBI. Multiple sequence alignments (clustalW method) and phylogenetic analysis were performed by the MEGA-X software (Version 10.1.8) (Kumar et al., 2018). The Jones-Taylor-Thornton (JTT) matrix-based model for a data set was chosen and the phylogenetic tree was constructed based on the alignment of the translated aa sequence by using the Maximum Likelihood method. The reliability of the analysis was assessed using a bootstrap test with 1000 replicates. Multiple alignments of the aa sequences were visualized using GeneDoc software (3/2/2007 updated version). Pairwise identity comparison analysis was implemented in SDT (Version 1.2) (Muhire et al., 2014) using the Muscle method and an R script was used for visualization.

## 3 Results

**Figure 1:**
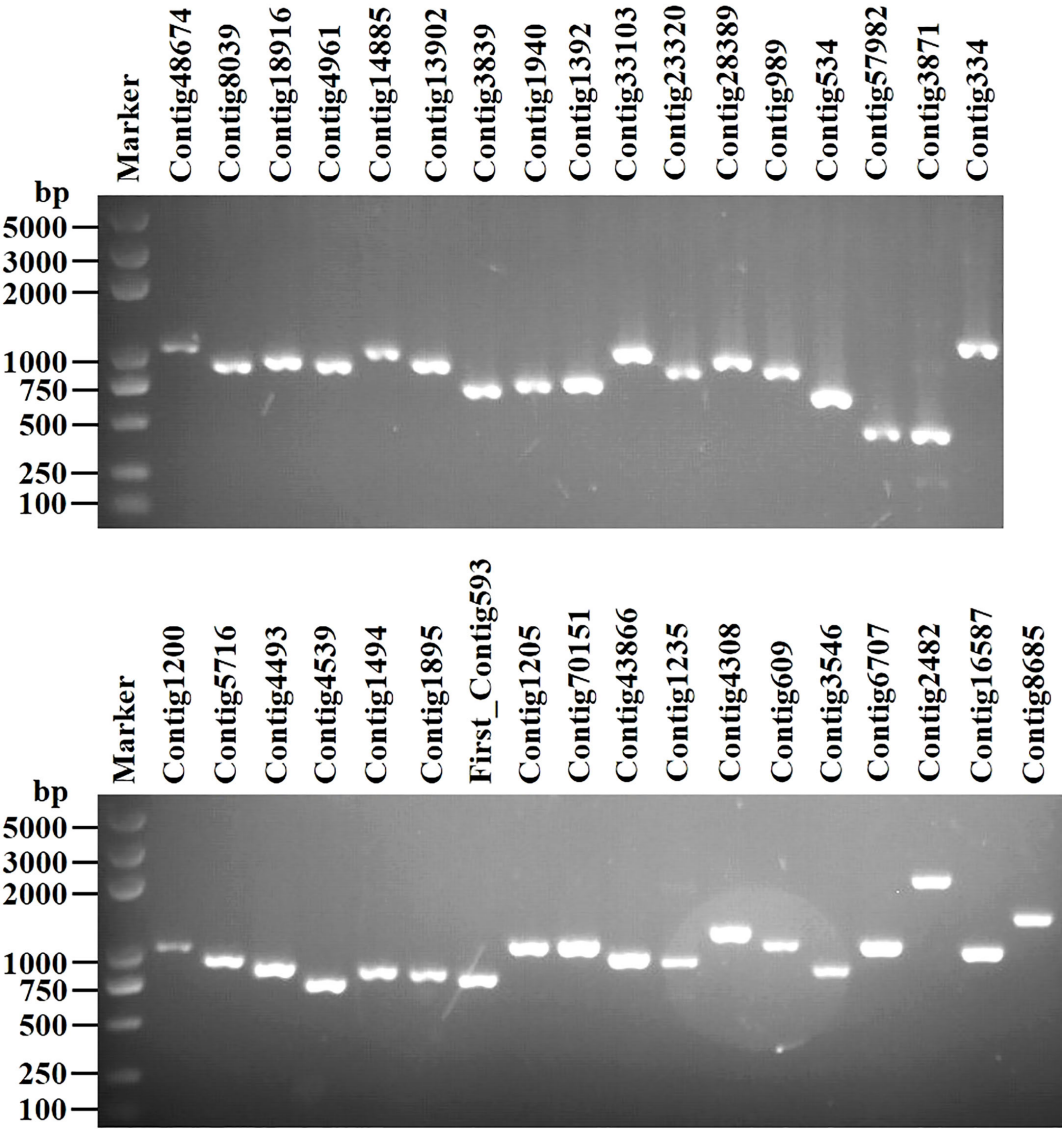
RT-PCR confirmation of 35 viral-like contigs in *Suillus luteus*. The viral primers were designed according to the obtained contig sequences. Primer pairs used and predicted sizes of amplicons are listed in **Table S2**. Lane Marker, DL2,000 bp DNA marker (Guangzhou Xinkailai Biotechnology Co., Ltd., China); Lane 1 to 35, obtained mycovirus contigs (see **Table 1** for details).

We identified 35 putative viral contigs representing 33 mycoviruses, 29 of which are novel viruses (**Table 1**). All sequences were subjected to the BLASTp analysis and phylogenetic analyses. Results suggest that these viruses can be grouped into 11 lineages, namely *Narnaviridae*, *Mitoviridae*, *Botourmiaviridae*, *Hypoviridae*, *Endornaviridae*, *Deltaflexiviridae*, *Megabirnaviridae*, *Alternaviridae*, *Mymonaviridae*, *Bunyaviriales*, and “*Mycoaspiviridae*”. Viruses with (+) ssRNA genomes (especially for members of the phylum *Lenarviricota*, including mycovirus families *Narnaviridae*, *Mitoviridae*, and *Botourmiaviridae*) are most prevalent in our study, accounting for 70% of all identified viruses. Whereas dsRNA and (-) ssRNA viruses are less frequent, accounting for 6% and 24%, respectively. RT-PCR amplification further demonstrated that these putative mycoviruses existed within these *S. luteus* isolates (**Figure 1**).

**Table 1:** Contigs with similarity to previously described viruses.

Contig number	GenBank accession	Contig length	Name of putative viruses	Abbreviation	Best match	aa identity	Family/Genus
Contig48674	OQ862536	2564	Suillus luteus narnavirus 1	SlNV1	Rhizopus oryzae narnavirus 1 (BDF97660.1)	0.33	*Narnaviridae*
Contig8039	OQ862537	2288	Suillus luteus narnavirus 2	SlNV2	Rhizopus oryzae narnavirus 1 (BDF97660.1)	0.48	*Narnaviridae*
Contig18916	OQ862538	2708	Suillus luteus narnavirus 3	SlNV3	Erysiphe necator associated narnavirus 42 (QJT93774.1)	0.36	*Narnaviridae*
Contig4961	OQ862540	1959	Suillus luteus narnavirus 4	SlNV4	Aspergillus flavus narnavirus 1 (UAW09567.1)	0.77	*Narnaviridae*
Contig14885	OQ862539	2140	Suillus luteus narnavirus 4	SlNV4	Aspergillus flavus narnavirus 1 (UAW09566.1)	0.73	*Narnaviridae*
Contig13902	OQ862544	2625	Suillus luteus narnavirus 5	SlNV5	Rhizopus microsporus 20S narnavirus (QBC65280.1)	0.83	*Narnaviridae*
Contig3839	OQ862541	2006	Suillus luteus narnavirus 6	SlNV6	Plasmopara viticola lesion associated narnavirus 7 (QIR30286.1)	0.41	*Narnaviridae*
Contig1940	OQ862542	2266	Suillus luteus narnavirus 7	SlNV7	Erysiphe necator associated narnavirus 37 (QJT93769.1)	0.45	*Narnaviridae*
Contig1392	OQ862543	2350	Suillus luteus narnavirus 8	SlNV8	Erysiphe necator associated narnavirus 49 (QJT93781.1)	0.79	*Narnaviridae*
Contig33103	OQ862545	2537	Suillus luteus mitovirus 1	SlMV1	Mitovirus sp. (MN034983.1)	0.44	*Mitoviridae*
Contig23320	OQ862546	2421	Suillus luteus mitovirus 2	SlMV2	Entomophthora muscae mitovirus 5 (BK010733.1)	0.45	*Mitoviridae*
Contig28389	OQ862547	2267	Suillus luteus mitovirus 3	SlMV3	Entomophthora muscae mitovirus 2(BK010730.1)	0.48	*Mitoviridae*
Contig989	OQ862548	2429	Suillus luteus mitovirus 4	SlMV4	Sclerotinia sclerotiorum mitovirus 39 (MT646411.1)	0.56	*Mitoviridae*
Contig534	OQ862549	2636	Suillus luteus mitovirus 5	SlMV5	Neofusicoccum parvum mitovirus 3 (MW175882.1)	0.64	Mitoviridae
Contig57982	OQ862550	2498	Suillus luteus mitovirus 6	SlMV6	Nigrospora oryzae mitovirus 2 (MH823902.1)	0.55	*Mitoviridae*
Contig3871	OQ862551	2601	Suillus luteus mitovirus 7	SlMV7	Fusarium mangiferae mitovirus 3 (MZ493903.1)	0.85	*Mitoviridae*
Contig334	OQ862552	2410	Suillus luteus mitovirus 8	SlMV8	Fusarium sambucinum mitovirus 5 (LC596829.1)	0.85	*Mitoviridae*
Contig1200	OQ862553	2741	Suillus luteus botourmiavirus 1	SlBV1	Botourmiaviridae sp. (WAK77894.1)	0.5	*Botourmiaviridae*
Contig5716	OQ862554	2586	Suillus luteus botourmiavirus 2	SlBV2	Hulunbuir Botou tick virus 4 (UYL95442.1)	0.98	*Botourmiaviridae*
Contig4493	OQ862555	2436	Suillus luteus botourmiavirus 3	SlBV3	Aspergillus pseudoviridinutans botourmiavirus 1 (BCH36653.1)	0.64	*Botourmiaviridae*
Contig4539	OQ862556	7600	Suillus luteus hypovirus 1	SlHV1	Alternaria dianthicola hypovirus 1 (UYZ32447.1)	0.65	*Hypoviridae*
Contig1494	OQ862557	14972	Suillus luteus hypovirus 2	SlHV2	Apis hypovirus 2 (UCR92524.1)	0.3	*Hypoviridae*
Contig1895	OQ862558	13673	Suillus luteus endornavirus 1	SlEV1	Rhizopus microspores endornavirus 1 (BDF97664.1)	0.38	*Endornaviridae*
First_Contig593	OQ862559	4881	Suillus luteus deltaflexivirus 1	SlDFV1	Rhizoctonia solani flexivirus 1 (ANR02698.1)	0.42	*Deltaflexiviridae*
Contig1205	OQ862560	8107	Suillus luteus megabirnavirus 1	SlMBV1	Sclerotinia sclerotiorum megabirnavirus 1 (YP_009143528/YP_009143529)	0.95/0.89	*Megabirnaviridae*
Contig70151	OQ862561	2129	Suillus luteus alternavirus 1	SlAV1	Diaporthe alternavirus 1 (BDQ13829.1)	0.5	*Alternaviridae*
Contig43866	OQ862562	2191	Suillus luteus alternavirus 1	SlAV1	Diaporthe alternavirus 1 (BDQ13830.1)	0.41	*Alternaviridae*
Contig1235	OQ862563	7071	Suillus luteus mymonavirus 1	SlMyV1	Xinjiang mymona-like virus 2 (QYF49867.1)	0.6	*Mymonaviridae*
Contig4308	OQ862564	7819	Suillus luteus associated bunya-like virus 1	SlaBV1	Ditton virus (AWA82278.1)	0.52	possible new family
Contig609	OQ862565	7847	Suillus luteus associated bunya-like virus 2	SlaBV2	Mucor phasmavirus A (QED42998.1)	0.95	possible new family
Contig3546	OQ862566	6568	Suillus luteus associated bunya-like virus 3	SlaBV3	Guyuan tick virus 1 (UYL95512.1)	0.63	*Discoviridae*
Contig6707	OQ862567	7887	Suillus luteus associated bunya-like virus 4	SlaBV4	Phasmaviridae sp. (WAK75648.1)	0.85	possible new family
Contig2482	OQ862568	6952	Suillus luteus associated bunya-like virus 5	SlaBV5	Botrytis cinerea negative-stranded RNA virus 6 (QJT73694.1)	0.38	“*Mycophleboviridae*”
Contig16587	OQ862569	4636	Suillus luteus associated bunya-like virus 6	SlaBV6	Erysiphe necator associated negative-stranded RNA virus 24 (QJW70356.1)	0.45	*“Mybuviridae”*
Contig8685	OQ862570	7244	Suillus luteus mycoophiovirus 1	SlMoV1	Plasmopara viticola lesion associated mycoophiovirus 5 (QJX19791.1)	0.46	“*Mycoaspiviridae*”

### 3.1 Eight novel viruses in the family *Narnaviridae*

Viruses in the family *Narnaviridae* typically possess a non-segmented linear (+) ssRNA genome ranging from 2.3 to 3.6 kb and contain a single ORF coding solely for the RdRp (Hillman and Cai, 2013). Only two species in this family, namely Saccharomyces 20S RNA narnavirus and Saccharomyces 23S RNA narnavirus, have been approved by the ICTV. However, the narnaviral phylogeny has been greatly expanded due to the discovery of numerous narnaviruses in various hosts (Akopyants et al., 2016; Shi et al., 2016; Grybchuk et al., 2018) with diverse genome structures (Chiapello et al., 2020b; Sutela et al., 2020; Kondo et al., 2022). This has resulted in increased complexity in taxonomy within this family. Dinan et al. have shown that the family *Narnaviridae* should at least comprise two genera, “*Alphanarnavirus*” and “*Betanarnavirus*” (Dinan et al., 2020), and further taxonomical studies are still undergoing (Chiapello et al., 2020a; Sutela et al., 2020; Sadiq et al., 2022). A total of 9 contigs were associated with members in the family *Narnaviridae*, indicating 8 distinctive narnaviruses discovered in *S. luteus*, provisionally named Suillus luteus narnavirus 1-8 (SlNV1-8) (**Table 1**).

The assembled sequences ranged from 1959 to 2708 nt in size, each of these sequences contains one ORF coding for the RdRp with 600-862 aa in length (**Table 1**). In addition, SlNV7 has an extra reverse ORF (rORF) on the negative-sense strand that encodes a hypothetical protein of 655 aa showing no homology to any known proteins. The same ambisense nature has also been reported in some narnaviruses (Chiapello et al., 2020a; Dinan et al., 2020). According to the BLASTp analysis, SlNV1 and 2 showed the highest similarity to the Rhizopus oryzae narnavirus 1 with identity at 33% and 48%, respectively (**Table 1**). SlNV3 was most similar to Erysiphe necator associated narnavirus 42 at 36% identity. The virus SlNV4 is comprised of two segments, and likely belonging to a newly defined group of narnaviruses called splipalmiviruses (Sutela et al., 2020). The RNA1 and RNA2 segments of SlNV4 display the greatest similarity to two segments of a tri-segmented narnavirus known as Aspergillus flavus narnavirus 1 (Degola et al., 2021), showing 77% and 73% identity, respectively. SlNV5 showed the highest similarity to Rhizopus microsporus 20S narnavirus with 83% identity. SlNV6 was most related to Plasmopara viticola lesion associated narnavirus 7 with 41% identity. Finally, SlNV7 and 8 were most similar to Erysiphe necator associated narnavirus 37 and 49 with 45% and 79% identity, respectively.

**Figure 2:**
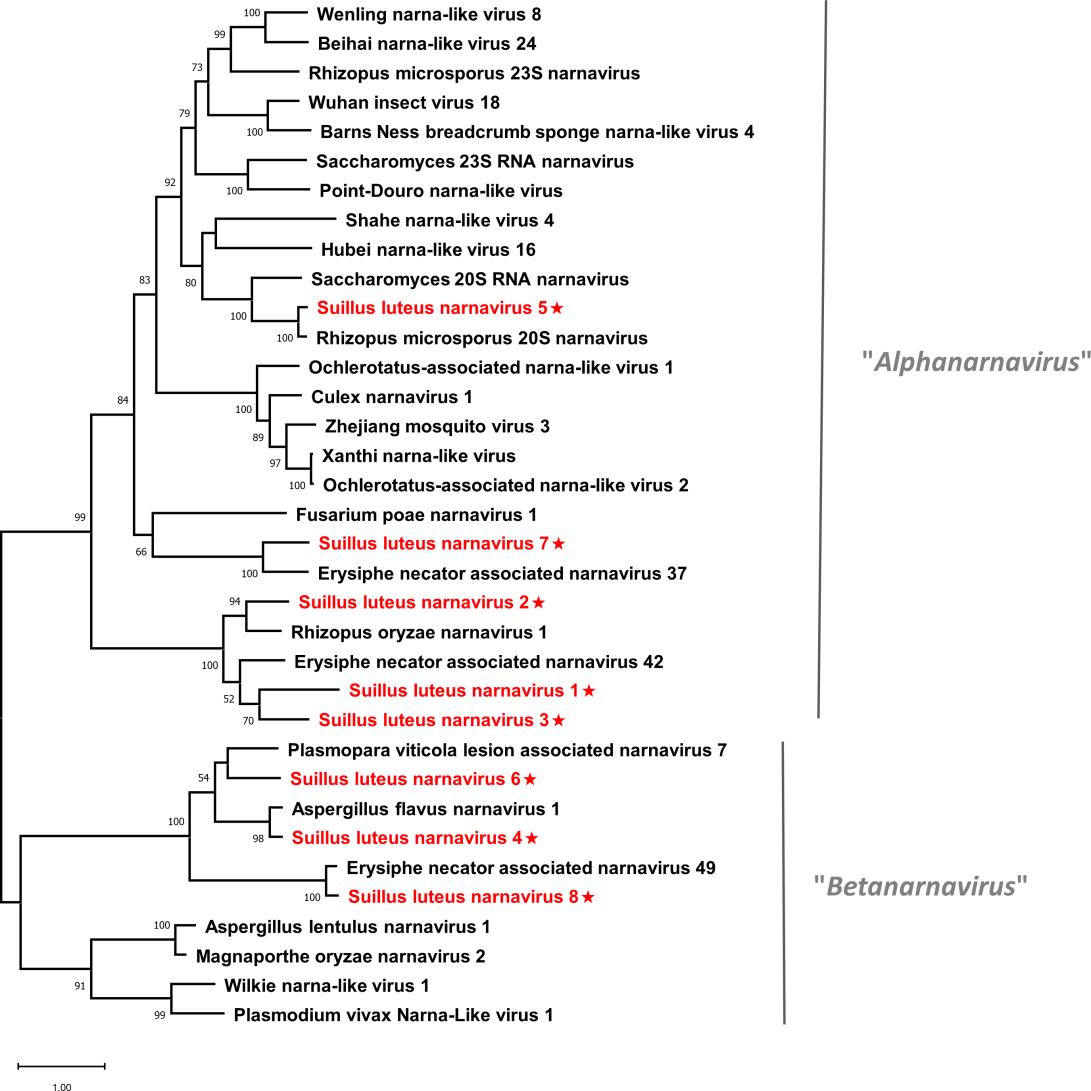
Unrooted phylogenetic tree constructed based on the alignment of RdRp amino acid sequences of members within the family *Narnaviridae*. The tree was constructed using the Maximum Likelihood method and the numbers at the nodes are evaluated by bootstrap analysis (1000 replicates). Only values above 50% are shown. All identified narnaviruses are highlighted in red and indicated with a star. The scale bar represents 1.0 amino acid substitutions per site. See **Table S1** for detailed information of each selected virus used to conduct the analysis.

Pairwise identity comparisons of narnaviruses reported in this study and other known narnaviruses were conducted (**Figure S1A**). The result indicated that no narnaviruses share sequence identity greater than 49% at the aa level and 62% at the nucleotide level with other narnaviruses, excluding SlNV4, -5, -8 which showed higher similarity to their best match in BLASTp analysis (>70% identity at both aa and nucleotide level). Following the eight conserved motifs indicated by a previous study (Koonin et al., 1991), the same motifs were also detected in SlNV1, -2, -3, -5, and -7 based on their alignment of RdRp sequences (**Figure S2A**). SlNV4, like other splipalmiviruses, possesses the motifs I to V on the protein encoded by RNA1 (**Figure S2B**) and motifs VI to VIII on the protein encoded by RNA2 (**Figure S2C**) (Sutela et al., 2020). The RdRp sequences of SlNV6 and 8 (**Figure S2A**) contain only motifs I to V, indicating that these sequences likely represent the RNA1 segment of splipalmiviruses. However, further research is needed to determine the RNA2 segment. In addition, a phylogenetic tree was constructed based on the complete RdRp aa sequence of identified and other selected narnaviruses (**Figure 2**). SlNV1, -2, -3, -5, and -7 seem to be phylogenetically related to the proposed genus “*Alphanarnavirus*”, while SlNV4, -6, -8 clustered with members of the proposed genus “*Betanarnaviruses*”.

### 3.2 Eight novel viruses and one characterized virus in the family *Mitoviridae*

Members in the family *Mitoviridae* have a mitochondrially replicating nature. Mitoviruses possess a monopartite linear (+) ssRNA genome that encodes only an RdRp, with a genome size of about 2.2-5.0 kb (Hillman and Cai, 2013). The family contains four genera, *Unuamitovirus*, *Duamitovirus*, *Triamitovirus*, and *Kvaramitovirus*. We have identified eight mitovirus-related contigs, representing the discovery of eight novel viruses within the family *Mitoviridae*, namely Suillus luteus mitovirus 1-8 (SlMV1-8) (**Table 1**).

All assembled sequences were 2267-2636 nt in length, likely to be the nearly complete genome of the corresponding viruses, which possess a complete ORF that encodes an RdRp ranging from 670 to 784 aa (**Table 1**). The BLASTp analysis showed that SlMV1 was most similar to an unnamed mitovirus reported in a soil metatranscriptomic study at 44% identity (Starr et al., 2019). SlMV2 and 3 showed the highest similarity to Entomophthora muscae mitovirus 5 and 2 (genus *Unuamitovirus*), with 45% and 48% identity, respectively. SlMV4 was most related to Sclerotinia sclerotiorum mitovirus 39 at 56% identity, while SlMV5 best matched Neofusicoccum parvum mitovirus 3 with 64% identity. SlMV6 showed the highest similarity to Nigrospora oryzae mitovirus 2 (genus *Unuamitovirus*) with 55% identity. In addition, SlMV7 and 8 showed significant levels of similarity (both 85% identity) to the mitoviruses in the *Fusarium mangiferae* and *F. sambucinum*, implying that SlMV7 and 8 may respectively represent different isolates of these known mitoviruses.

**Figure 3:**
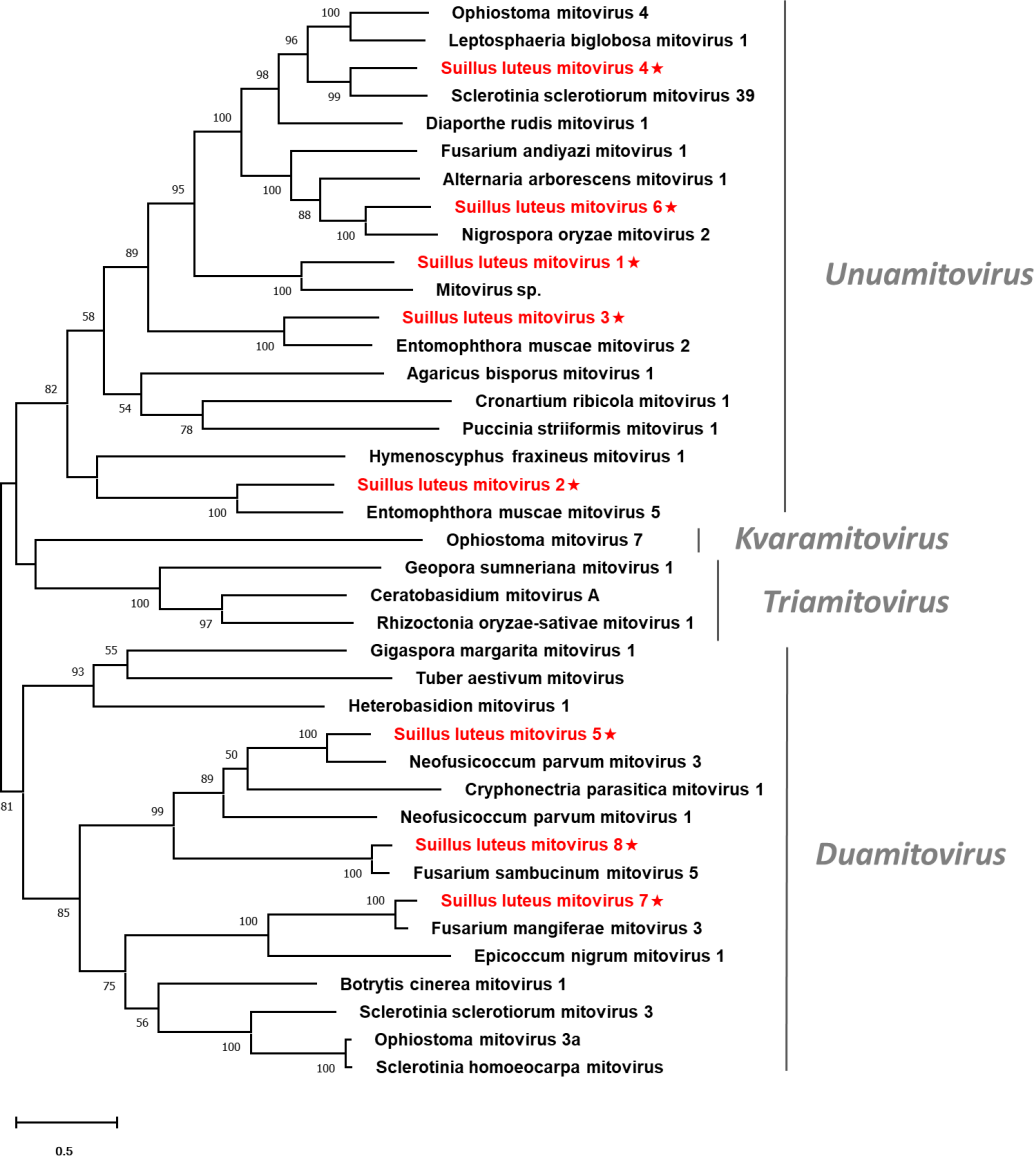
Unrooted phylogenetic tree constructed based on the alignment of RdRp amino acid sequences of members within the family *Mitoviridae*. The tree was constructed using the Maximum Likelihood method and the numbers at the nodes are evaluated by bootstrap analysis (1000 replicates). Only values above 50% are shown. All identified mitoviruses are highlighted in red and indicated with a star. The scale bar represents 0.5 amino acid substitutions per site. See **Table S1** for detailed information of each selected virus used to conduct the analysis.

Pairwise identity comparisons were conducted based on mitoviruses reported in this study and other mitoviruses (**Figure S1B**). The result suggested that no characterized mitoviruses share sequence identity greater than 64% at the aa level and 70% at the nucleotide level to other mitoviruses, except SlMV7 and 8 that showed the highest similarity to their best match in BLASTp analysis (86% and 85% at the aa level). Multiple alignments of the RdRp of the eight identified mitoviruses and other selected mitoviruses detected six conserved motifs, which is characteristic of mitoviruses (**Figure S3**) (Hong et al., 1999). To establish the phylogeny of the identified mitoviruses and other selected mitoviruses, a phylogenetic tree was constructed based on the RdRp proteins. The result showed that SlMV1, -2, -3, -4, -6 are clustered with members of the genus *Unuamitovirus*, and SlMV5, -7, -8 are clustered with members within the genus *Duamitovirus* (**Figure 3**).

### 3.3 Two novel viruses and one characterized virus in the family *Botourmiaviridae*

The family *Botourmiaviridae* is composed of twelve genera, eleven of which infect fungi. Members in these fungi-infecting genera encompass nonsegmented linear (+) ssRNA genomes ranging from 2 kb to 5.3 kb and only encode an RdRp (Sadiq et al., 2022). Three contigs associated with members of the family *Botourmiaviridae* were identified, demonstrating three novel botourmiaviruses were characterized in the fungus *S. luteus*. We named them as Suillus luteus botourmiavirus 1, 2, and 3 (SlBV1, -2, and -3) (**Table 1**).

SlBV1 possesses a nearly complete genome of 2741 nt and encodes a putative RdRp protein of 646 aa, which showed the highest similarity to an unnamed botourmiavirus hosted in *Haemaphysalis longicornis* with 50% identity. SlBV2 had a complete ORF encoding a putative RdRp of 662 aa. The BLASTp analysis showed that the putative protein was almost identical to the RdRp of Hulunbuir Botou tick virus 4 (HBTV4) at 98% identity, suggesting that SlBV2 is likely an isolate of HBTV4. SlBV3 had a 2436-nt genome with a complete ORF that encodes a 639-aa RdRp. BLASTp analysis showed that this protein best matches the RdRp of Aspergillus pseudoviridinutans botourmiavirus 1 with 64% identity.

**Figure 4:**
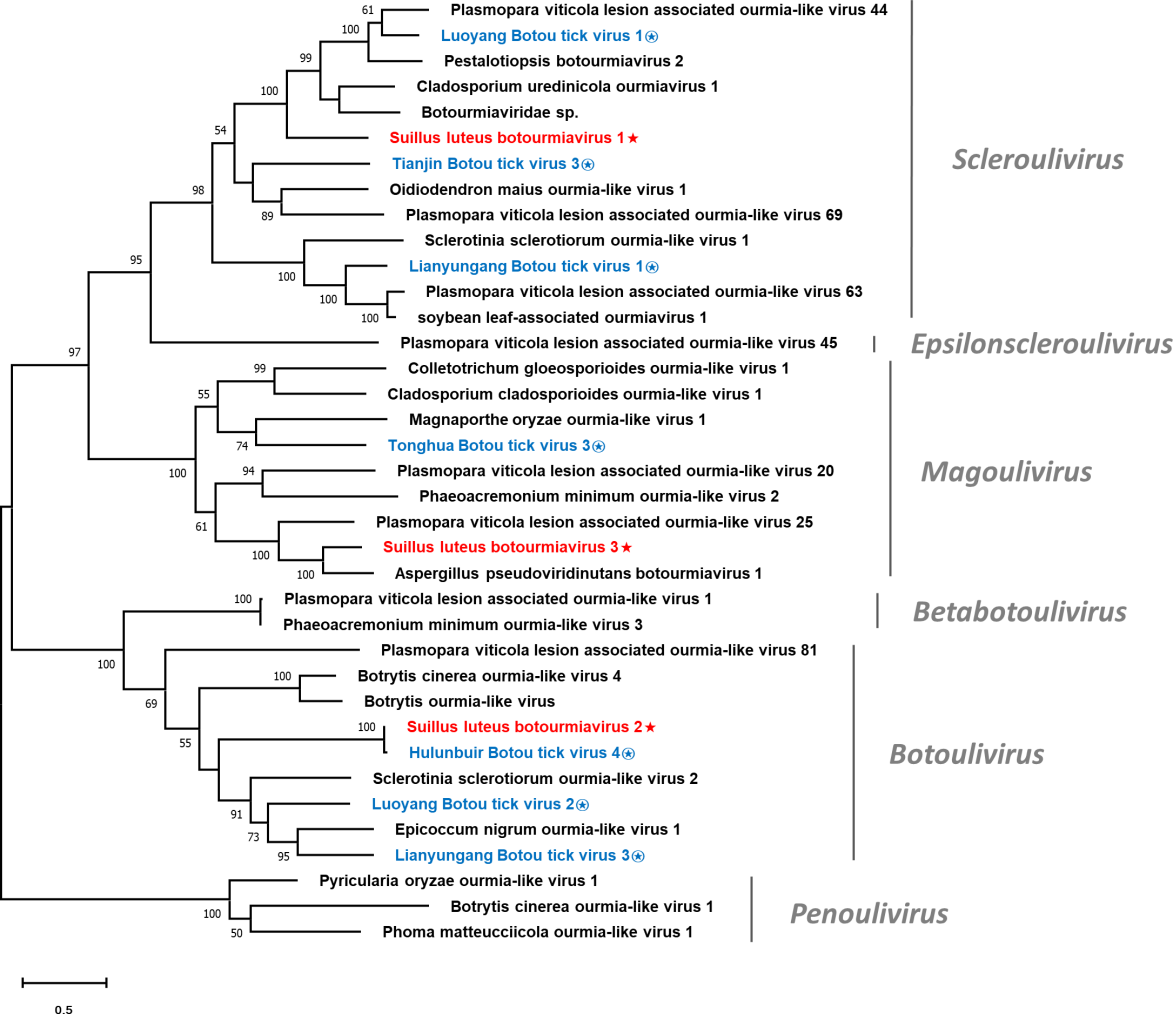
Unrooted phylogenetic tree constructed based on the alignment of RdRp amino acid sequences of members within the family *Botourmiaviridae*. The tree was constructed using the Maximum Likelihood method and the numbers at the nodes are evaluated by bootstrap analysis (1000 replicates). Only values above 50% are shown. All identified botourmiaviruses are highlighted in red and indicated with a star, while all botourmiaviruses that are hosted in ticks are highlighted in blue and indicated with a circled star. The scale bar represents 0.5 amino acid substitutions per site. See **Table S1** for detailed information of each selected virus used to conduct the analysis.

Multiple alignments of the RdRp of SlBV1, SlBV2, SlBV3, and other selected botourmiaviruses were conducted (**Figure S4**), supporting the existence of eight conserved motifs that were characterized in the RdRps of (+) ssRNA mycoviruses (Koonin, 1991). Phylogenetic analysis for the Suillus botourmiaviruses and other selected botourmiaviruses was conducted (**Figure 4**). The well-supported result revealed that SlBV1, SlBV2, and SlBV3 are clustered with members of *Scleroulivirus*, *Botoulivirus*, and *Magoulivirus*, respectively.

### 3.4 Two novel viruses in the family *Hypoviridae*

The family *Hypoviridae* currently comprises eight newly-established genera, whose members usually possess (+) ssRNA genomes ranging from 9 to 13 kb. Hypoviruses contain at least one large ORF. The encoded polyprotein with protease, RdRp, and helicase domains are conserved in all hypoviruses (Suzuki et al., 2018). Two contigs showed similarity to viruses of the family *Hypoviridae* and likely represent two different species, which we tentatively named Suillus luteus hypovirus 1 and 2 (SlHV1 and 2) (**Table 1**).

SlHV1 had a partial ORF that encodes a 2307-aa putative polyprotein, showing the highest similarity to the polyprotein of Alternaria dianthicola hypovirus 1 with 65% identity. According to the CD search on NCBI, this polyprotein contained UDP-glycosyltransferase (UGT), peptidase, and RdRp domains. SlHV2 had a nearly complete genome of 14972 nt, which possesses an ORF coding for a polyprotein of 4481 aa. The Blastp analysis revealed that this polyprotein was most similar to that of the Apis hypovirus 2 with 30% identity. Two conserved domains (RdRp and helicase) were detected in this polyprotein.

**Figure 5:**
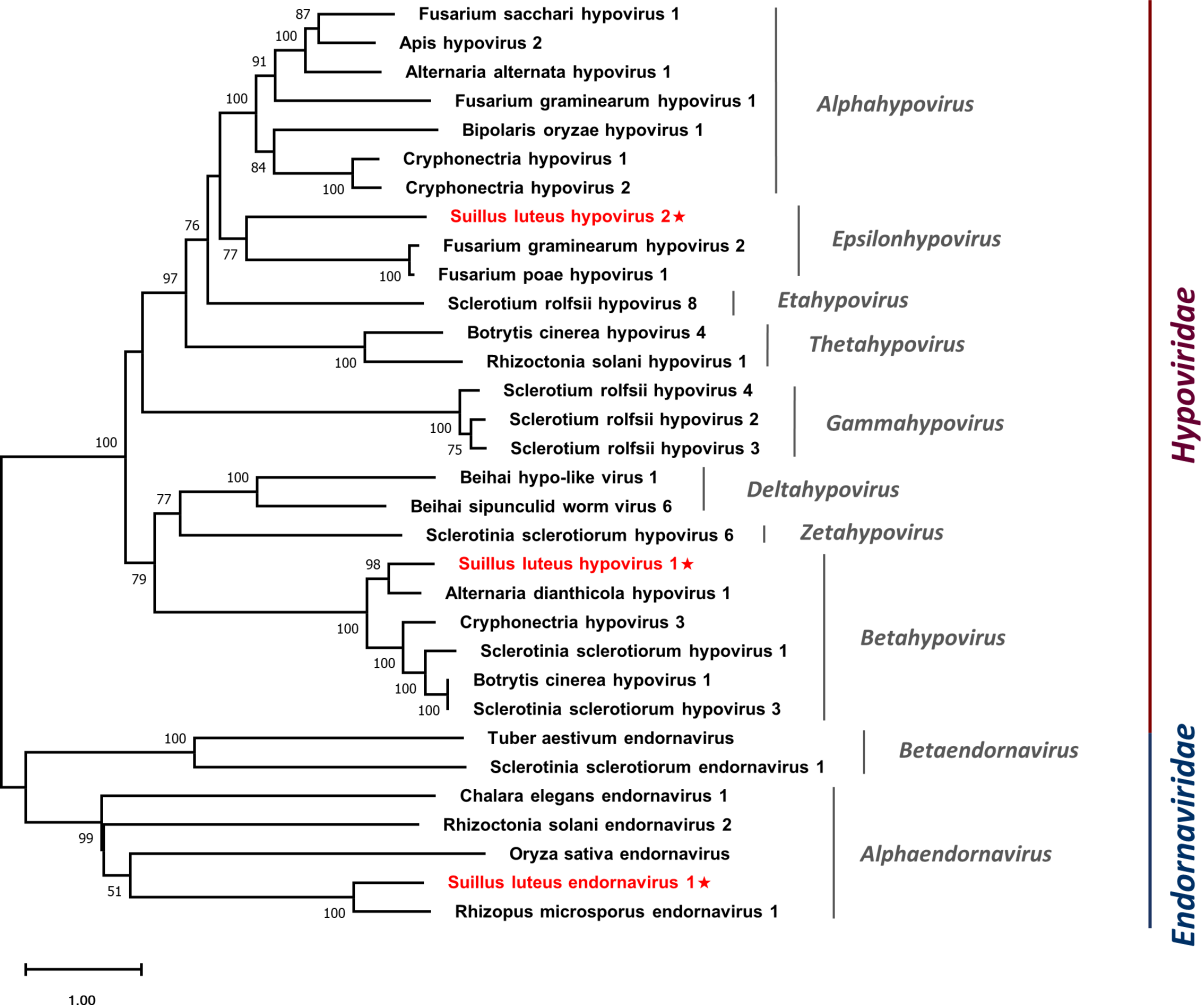
Unrooted phylogenetic tree constructed based on the alignment of RdRp amino acid sequences of identified hypoviruses and endornavirus, together with other selected viruses. The tree was constructed using the Maximum Likelihood method and the numbers at the nodes are evaluated by bootstrap analysis (1000 replicates). Only values above 50% are shown. All identified viruses are highlighted in red and indicated with a star. The scale bar represents 1.0 amino acid substitutions per site. See **Table S1** for detailed information of each selected virus used to conduct the analysis.

Multiple alignments were conducted based on the aa sequences of SlHV1, SlHV2, and other selected hypoviruses. Nine conserved motifs were characterized in the hypoviral RdRps (**Figure S5A**) (Koonin et al., 1991), and seven motifs were detected in the helicase domain (**Figure S5B**) (Koonin et al., 1993). In addition, a phylogenetic tree was constructed based on the polyprotein of identified hypoviruses and other selected hypoviruses (**Figure 5**). The result suggests that SlHV1 is more closely related to members of the genus *Betahypovirus*, while SlHV2 seems to be closer to the genus *Epsilonhypovirus*.

### 3.5 A novel virus in the family *Endornaviridae*

Members of the family *Endornaviridae* are capsidless viruses with (+) ssRNA genomes of 9.7-17.6 kb and contain a single ORF that encodes a polyprotein ranging from 3200 to 5800 aa with helicase and RdRp domains (Valverde et al., 2019). Endornaviruses are classified into two genera, namely *Alphaendornavirus* and *Betaendornavirus*. A contig showing similarity to the members of the family *Endornaviridae* was identified, suggesting the characterization of a novel virus named Suillus luteus endornavirus 1 (SlEV1) (**Table 1**).

SlEV1 had a nearly complete genome of 13673 nt and encoded a polyprotein of 4534 aa. The polyprotein was most similar to that of Rhizopus microsporus endornavirus 1 at 38% identity. Using the CD search on NCBI, RdRp and helicase domains were detected in this polyprotein.

Multiple alignments of the aa sequences of SlEV1 and other selected endornaviruses were conducted, eight motifs were detected in the RdRp domain (**Figure S6A**) (Koonin et al., 1993) and six motifs were detected in the endornaviral helicase domain (**Figure S6B**) (Hacker et al., 2005). A phylogenetic analysis was conducted based on the polyprotein of SlEV1 and selected viruses (**Figure 5**). The SlEV1 was clustered with members of the genus *Alphaendornavirus*, demonstrating SlEV1 should be a novel virus of this genus.

### 3.6 A novel virus in the family *Deltaflexiviridae*

The family *Deltaflexiviridae* comprises only one fungi-infecting genus, *Deltaflexivirus*. Members of this family have a genome of 8.1-8.3 kb in size with four to five ORFs (Bejerman and Debat, 2022). The largest ORF encodes a replication-associated polyprotein which usually contains three domains, including the methyltransferase, helicase, and RdRp (Martelli et al., 2007). First_Contig593 showed similarity to members of the family *Deltaflexiviridae* and likely represents a new species in this family, namely Suillus luteus deltaflexivirus 1 (SlDFV1) (**Table 1**).

SlDFV1 had a partial genome of 4881 nt, containing an ORF that encodes an incomplete polyprotein of 1576 aa. The CD search revealed RdRp, methyltransferase (Mtr), and helicase domains in this polyprotein. The polyprotein showed the highest similarity to Rhizoctonia solani flexivirus 1 with 42% identity.

**Figure 6:**
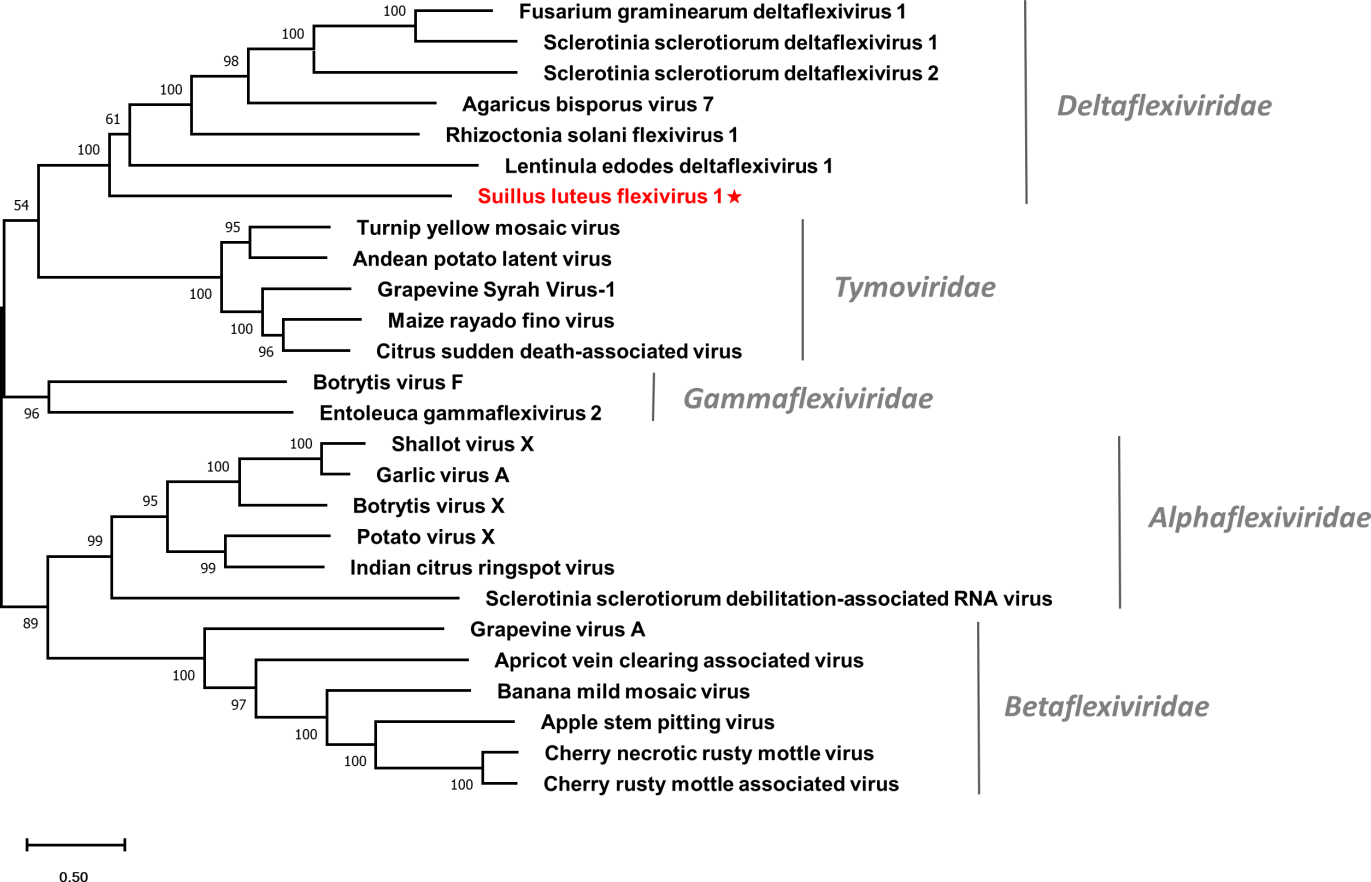
Unrooted phylogenetic tree constructed based on the alignment of RdRp amino acid sequences of identified flexiviruses and other selected viruses within the order *Tymovirales*. The tree was constructed using the Maximum Likelihood method and the numbers at the nodes are evaluated by bootstrap analysis (1000 replicates). Only values above 50% are shown. All identified viruses are highlighted in red and indicated with a star. The scale bar represents 0.5 amino acid substitutions per site. See **Table S1** for detailed information of each selected virus used to conduct the analysis.

Multiple alignments of the RdRp domain (**Figure S7A**) and helicase domain (**Figure S7B**) based on SlDFV1 and other selected deltaflexivirus were conducted. Six conserved motifs were detected in the RdRp domain and helicase domain, which is similar to the previous reports (Li et al., 2016). A phylogenetic tree was constructed for members of the order *Tymovirales* (**Figure 6**). The SlDFV1 clustered with members of the family *Deltaflexiviridae*, supporting the idea that SlDFV1 should be a new member of this family.

### 3.7 An isolate of a characterized megabirnavirus

The family *Megabirnaviridae* comprises only one genus, *Megabirnavirus*. Members of this family are non-enveloped spherical viruses with bi-segmented dsRNA genomes (dsRNA1 and dsRNA2). The dsRNA1 contains ORF1 and 2, which encodes capsid protein (CP) and RdRp, respectively. While ORF3 and 4 on dsRNA2 encode hypothetical proteins with unknown functions (Sato et al., 2019).

Contig1205 is 8107 nt in length and harbors two ORFs encoding the CP and RdRp, corresponding to ORF1 and ORF2 of Sclerotinia sclerotiorum megabirnavirus 1 (SsMBV1) with 95% and 89% identity, respectively. This suggested Contig1205 should represent an isolate of SsMBV1, specifically referred to as Suillus luteus megabirnavirus 1 (SlMBV1) (**Table 1**).

**Figure 7:**
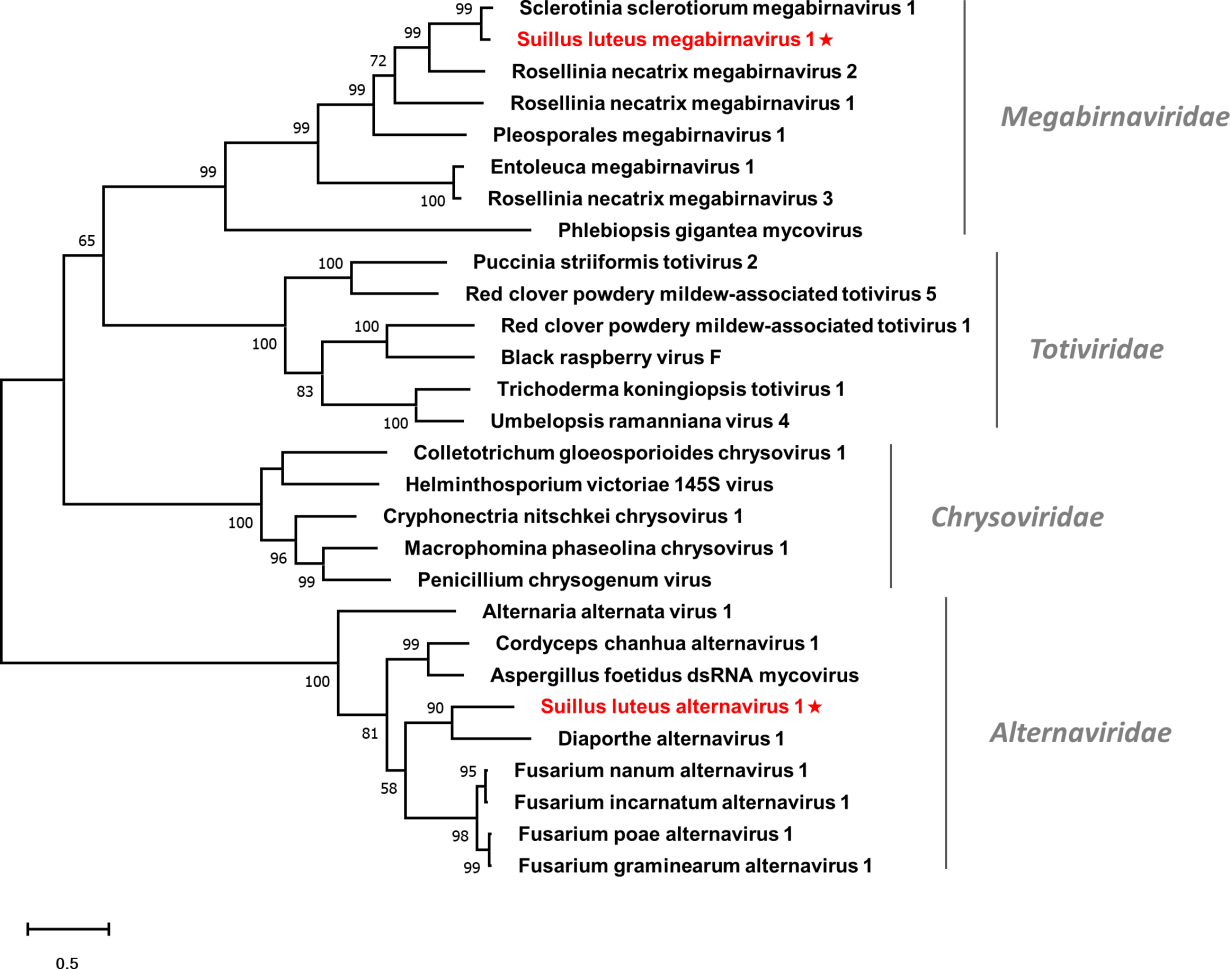
Unrooted phylogenetic tree constructed based on the alignment of RdRp amino acid sequences of identified dsRNA mycoviruses with other previously described viruses. The tree was constructed using the Maximum Likelihood method and the numbers at the nodes are evaluated by bootstrap analysis (1000 replicates). Only values above 50% are shown. All identified viruses are highlighted in red and indicated with a star. The scale bar represents 0.5 amino acid substitutions per site. See **Table S1** for detailed information of each selected virus used to conduct the analysis.

Multiple alignments of the RdRp domain detected eight conserved motifs (**Figure S8**), which is in agreement with a previous report (Wu et al., 2012). A phylogenetic tree based on the RdRp of SlMBV1 and other selected viruses was constructed (**Figure 7**). SlMBV1 clustered with other members of this family with well-supported bootstrap, suggesting that SlMBV1 should be a member of *Megabirnaviridae*.

### 3.8 A novel member of the family *Alternaviridae*

Members of the family *Alternaviridae* have a genome consisting of at least three dsRNA segments. Each dsRNA contains only one ORF. The largest dsRNA contains an ORF encoding the RdRp (Zhang et al., 2022), while in some alternaviruses, other dsRNA may encode a CP or a hypothetical protein with an unknown function. Two contigs showed similarity to viruses of this family (Kozlakidis et al., 2013) and are likely to represent a new member of this family, which we tentatively named Suillus luteus alternavirus 1 (SlAV1) (**Table 1**).

Contig70151 was 2129 nt in length, encoding a partial RdRp of 698 aa, while Contig43866 was 2191 nt in length, including an incomplete ORF that encodes a hypothetical protein of unknown function. Both RdRp and hypothetical protein were most similar to that of the Diaporthe alternavirus 1 with 50% and 41% identity respectively. Thus, these contigs may represent different RNA segments of SlAV1.

Multiple alignments were conducted based on the RdRp of SlAV1 and other selected alternavirus (**Figure S9**). Six out of all the eight conserved motifs were detected, while the other two motifs were missed due to the incomplete assembling of reads. Notably, the glycine residue of the GDD catalytic triad within motif VI was replaced by an alanine, which is also characterized in other alternavirus (Gilbert et al., 2019). Furthermore, a phylogenetic analysis showed that SlAV1 was clustered with members of the family *Alternaviridae* (**Figure 7**), giving evidence of the idea that SlAV1 should be a novel virus in this family.

### 3.9 A novel virus in the family *Mymonaviridae*

Viruses in the family *Mymonaviridae* have non-segmented (-) ssRNA genomes. The typical mymonavirus genome ranging from 6.2 to 11.6 kb is predicted to have four to seven non-overlapping ORFs, coding for RdRp that is essential to all members of this family, hypothetical proteins of unknown function, and nucleoprotein that encapsidates the mymonavirus genome (Jiāng et al., 2022). The mymonavirus genome has no poly(A) tail structure at the 3’ end. Contig1235 showed similarity to the family *Mymonaviridae*, possibly representing a new species in this family, termed Suillus luteus mymonavirus 1 here (SlMyV1) (**Table 1**).

Contig1235 was 7071 nt in length, containing three major ORFs. The biggest ORF encoding an RdRp of 1958 aa most similar to the RdRp of Xinjiang mymona-like virus 2 with 60% identity, whereas the other two ORFs were much smaller and coding for two hypothetical proteins showing no homology to any known proteins.

**Figure 8:**
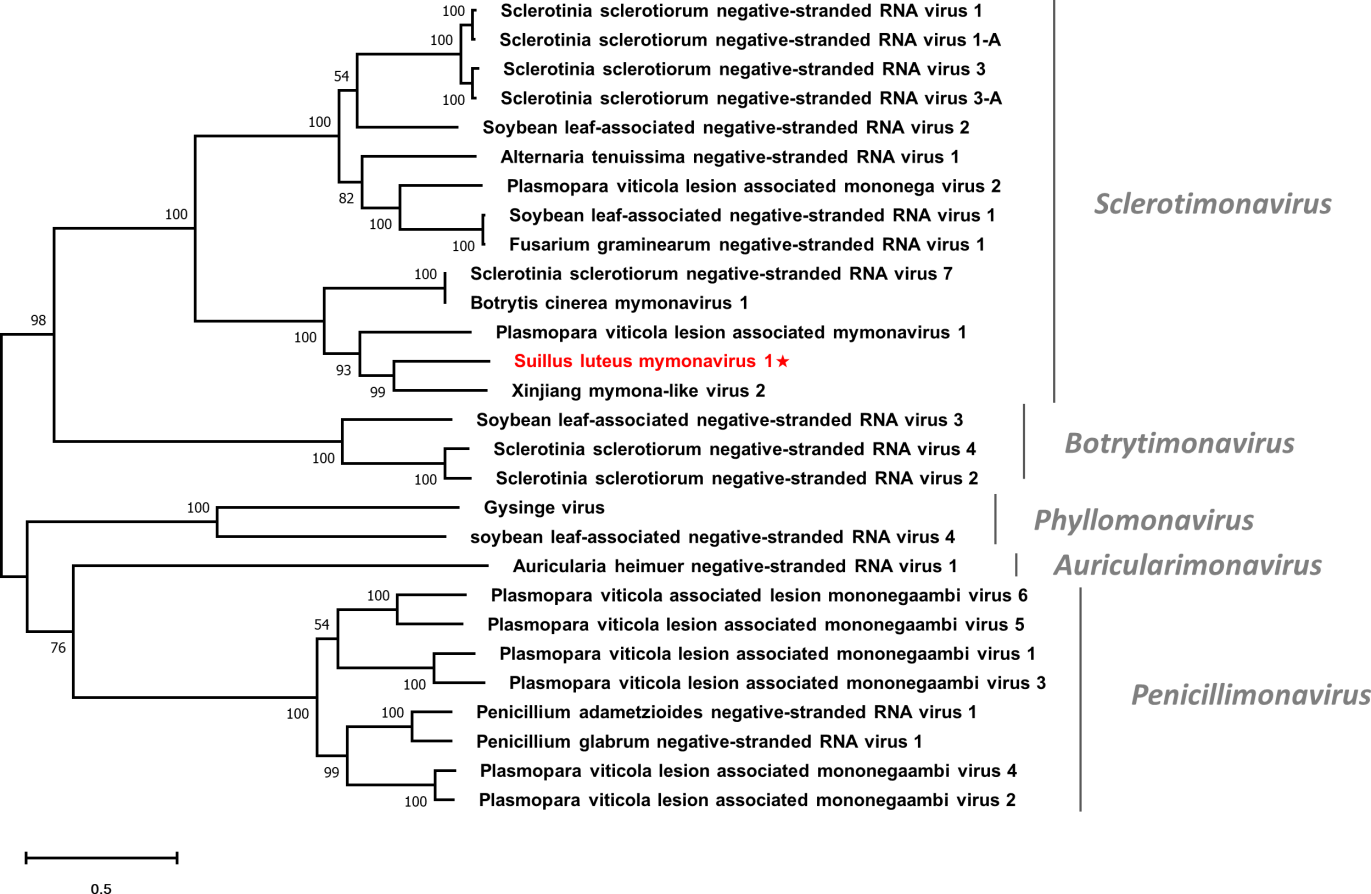
Unrooted phylogenetic tree constructed based on the alignment of RdRp amino acid sequences of members within the family *Mymonaviridae*. The tree was constructed using the Maximum Likelihood method and the numbers at the nodes are evaluated by bootstrap analysis (1000 replicates). Only values above 50% are shown. All identified viruses are highlighted in red and indicated with a star. The scale bar represents 0.5 amino acid substitutions per site. See **Table S1** for detailed information of each selected virus used to conduct the analysis.

Multiple alignments based on the RdRp of SlMyV1 and other selected mymonaviruses were conducted (**Figure S10**). Four conserved motifs that were identified in other mymonaviruses were also detected in the RdRp of SlMyV1 (Hao et al., 2018; Wang et al., 2022). In addition, phylogenetic analysis revealed that SlMyV1 clustered with members of the genus *Sclerotimonavirus* (**Figure 8**), suggesting SlMyV1 should be a new member of this genus.

### 3.10 Four novel viruses and two characterized virus in the order *Bunyavirales*

The *Bunyavirales* is an order of RNA viruses that contain a genome of 2-8 (-) ssRNA segments. The order *Bunyavirales* contains 14 families, among which three families accommodate mycoviruses, including three approved families, namely *Discoviridae*, *Phenuiviridae*, and *Tulasviridae*. More proposed families that are not formally approved by ICTV, such as “*Mycophleboviridae*”, “*Mybuviridae*”, “*Sclerobunyaviridae*”, and “*Mycobunyaviridae*”, also infect fungi (Picarelli et al., 2019; Jia et al., 2021; Ruiz-Padilla et al., 2021). Six contigs showed similarity to viruses of the order *Bunyavirales*, representing six species within this order, namely Suillus luteus associated bunya-like virus 1-6 (SlaBV1-6) (**Table 1**).

The sequences of identified viruses ranged from 4636 to 7887 nt in length and contained an ORF that encodes the RdRp of 1494-2572 aa. All sequences seem to be the nearly complete segments containing the ORF that encodes the RdRp of the corresponding viruses, except SlaBV6 which possesses an incomplete ORF. The BLASTp analysis revealed that SlaBV1, -2, and -4 were most similar to members of the family *Phasmaviridae*, a family of arthropod viruses within the order *Bunyavirales* (Li et al., 2015), with the identity of 52%, 95%, and 85%, respectively. While SlaBV3 was most similar to the Guyuan tick virus 1, a virus of *Peribunyaviridae*, with 63% aa identity. SlaBV5 and 6 showed similarity to two unclassified bunyaviruses isolated from different fungal hosts, namely Botrytis cinerea negative-stranded RNA virus 6 and Erysiphe necator associated negative-stranded RNA virus 24, with 38% and 45% identity, respectively.

**Figure 9:**
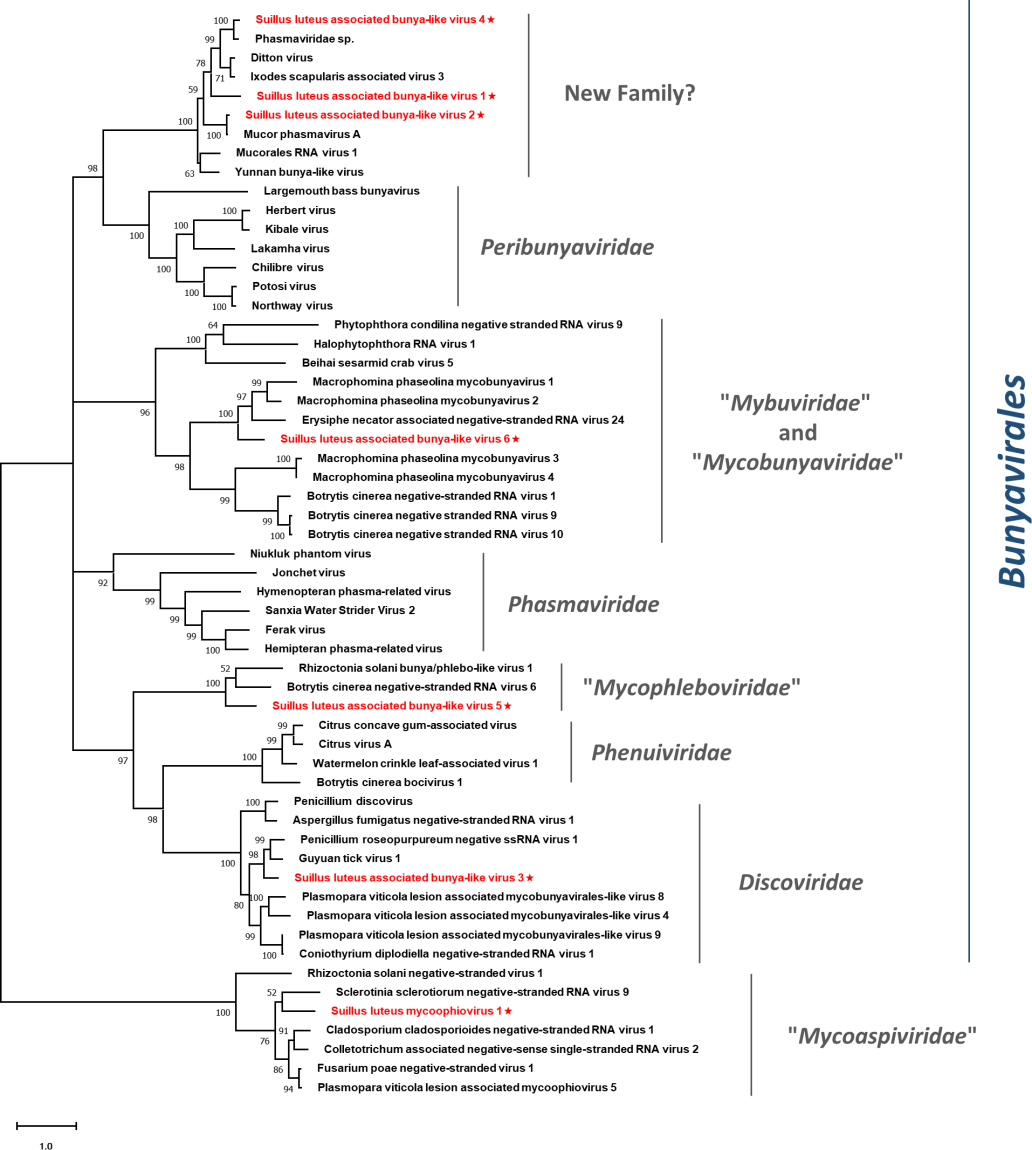
Unrooted phylogenetic tree constructed based on the alignment of RdRp amino acid sequences of identified mycoviruses with segmented (-) ssRNA genomes, together with other selected viruses. The tree was constructed using the Maximum Likelihood method and the numbers at the nodes are evaluated by bootstrap analysis (1000 replicates). Only values above 50% are shown. All the identified viruses are highlighted in red and indicated with a star. The scale bar represents 1.0 amino acid substitutions per site. See **Table S1** for detailed information of each selected virus used to conduct the analysis.

Pairwise identity comparisons of identified bunya-like viruses and other viruses within this order were conducted (**Figure S1C**). The result demonstrated that no identified bunya-like viruses share sequence identity greater than 64% at the aa level and 68% at the nucleotide level with other bunya-like viruses, excluding SlaBV2 and 4 which showed higher similarity to their best match in BLASTp analysis (>80% identity at both aa and nucleotide level). Multiple alignments of identified bunya-like viruses and other viruses within this order were conducted (**Figure S11**), detecting six conserved motifs that are typical for the RdRp of the viruses within the order *Bunyavirales* (Chen et al., 2021b). In addition, a phylogenetic tree was constructed based on the RdRp sequences and other selected viruses (**Figure 9**). The result suggested that SlaBV1, -2, and -4 belong to a novel family that includes both mycovirus and viruses of arthropods (including some viruses previously thought as members of the family *Phasmaviridae*). It is worth noting that members of the proposed “*Mybuviridae*” and “*Mycobunyaviridae*” did not form two distinct clades in our study, but showed great affinity to each other. SlaBV6 is phylogenetically related to this mixed clade. SlaBV5 is closely related to the proposed family “*Mycophleboviridae*”, and SlaBV3 is possibly a new member of *Discoviridae*.

### 3.11 A novel mycovirus in the proposed family “*Mycoaspiviridae*”

Members of the “*Mycoaspiviridae*” have a (-) ssRNA genome coding for an Ophiovirus-related RdRp. Despite the fact that the majority of mycoophioviruses have a single genome segment that encodes only RdRp, a previous investigation has revealed the presence of an extra genome segment in a particularly identified mycoophiovirus (Hamim et al., 2022). This discovery indicates the possibility that more mycoophioviruses might have extra segments yet to be confirmed, just consistent with Ophioviruses whose members are hosted in plants and also have segmented genomes. Contig8685 showed similarity to a newly proposed family “*Mycoaspiviridae*” (Chiapello et al., 2020a), and likely represents a new species in this family, namely Suillus luteus mycoophiovirus 1 (SlMoV1) (**Table 1**).

SlMoV1 had a nearly complete genome of 7244 nt, encoding an RdRp of 2346 aa. The BLASTp analysis showed that the RdRp was most similar to the Plasmopara viticola lesion associated mycoophiovirus 5 with 46% identity.

Multiple alignments of SlMoV1 and other mycoophioviruses detected all the five conserved motifs shared by mycoophioviruses (**Figure S12**) (Hamim et al., 2022). Phylogenetic analysis revealed that SlMoV1 clustered with members of the “*Mycoaspiviridae*” (**Figure 9**). Thus SlMoV1 should be a new member of this family.

## 4 Discussion and conclusion

Metagenomics has greatly enriched the understanding of virus diversity and gradually uncovered the evolutionary mysteries of RNA viruses (Dolja and Koonin, 2018; Sadiq et al., 2022). In this study, we utilized NGS technology to investigate the virome of *S. luteus*. With the assistance of BLASTp analysis and phylogenetic tree construction, 35 putative viral sequences representing 33 mycoviruses were identified, 29 of which are novel viruses. The mycoviruses identified had a diversity of genomes, and can be grouped into 3 groups, including six families with (+) ssRNA genomes, two families with dsRNA genomes, and three lineages of (-) ssRNA viruses. It should be noted that the genome forms of these viruses were only inferred from their lineages since all contigs were obtained through cDNA sequencing. Thus, the actual forms of their genomes require further confirmation.

### 4.1 Further biodiversity of (+) ssRNA mycoviruses

The fungal virosphere was once believed to be dominated by dsRNA viruses. However, the increasing application of NGS has led to the discovery of a significant number of (+) ssRNA viruses that exist within the fungal kingdom (Kondo et al., 2022). Viruses with (+) ssRNA genomes are most abundant in our study, accounting for 71% of all identified viruses. These viruses can be classified into six families, *Narnaviridae*, *Mitoviridae*, *Botourmiaviridae*, *Hypoviridae*, *Endornaviridae*, and *Deltaflexiviridae*. The members within the phylum *Lenarviricota* are the most prevalent viruses not only in our study (59% of all identified viruses), but also in other similar virome studies (Mu et al., 2017; Ruiz-Padilla et al., 2021; He et al., 2022). It is one of the only two viral phyla that include both prokaryote- and eukaryote-infecting members (Kondo et al., 2022), making it an interesting topic for investigating the evolutionary trajectories of RNA viruses (Shi et al., 2016; Wolf et al., 2018). The phylum is known for its diverse collection of (+) ssRNA viruses, with the family *Narnaviridae* exhibiting noteworthy variety in genomic architecture and host range. The authentic narnaviruses, which were considered to be the simplest viruses (Hillman and Cai, 2013), have an unsegmented linear (+) ssRNA genome containing a single ORF that encodes solely for the RdRp. However, the narnaviral phylogeny has been significantly expanded due to recent studies that have uncovered unprecedented genomes.

One example that was identified during our research is the group of narnaviruses containing rORF on their negative strand. A previous study has indicated that narnaviruses with rORF exhibit a distinctive tendency to avoid using reverse complements of stop codons (CUA, UUA, and UCA) in their RdRp coding ORF, and instead prefer to use alternative synonymous codons as substitutes (Dinan et al., 2020). Although protein products of these rORFs revealed high divergence in aa sequences (Chiapello et al., 2020a; Dinan et al., 2020), it is likely that the rORF is functional and confers an evolutionary benefit, as evidenced by its selection for maintenance. Further study will be required to characterize the possible function of these hypothetical proteins.

Another group of narnaviruses known as splipalmiviruses was also detected in our study. These viruses possess divided RdRp palm domains, which are encoded separately by two genomic segments (Sutela et al., 2020; Kondo et al., 2022). Whereas some splipalmiviruses have been identified to possess additional segments with unknown function (Chiba et al., 2021a; Degola et al., 2021; Jia et al., 2021). The formation of RdRp complex has been proven by a homology modeling study, which demonstrated that the two RdRp fragments can bind together through hydrogen bonds and van der Waals forces (Chiba et al., 2021a). Furthermore, a unique narnavirus, termed Aspergillus tennesseensis narnavirus 1 (AtenNV1), was found to have a different division site in RdRp and formed a distinct phylogenetic clade with splipalmiviruses (Chiba et al., 2021b). This discovery provides evidence for independent splitting events of RdRp occurring within *Narnaviridae*. Split RdRp domains are exclusively found in splipalmiviruses and AtenNV1 among RNA virosphere (Kondo et al., 2022). It is reasonable to assume that there may be a wider range of split RdRp RNA viruses than we previously thought, given the independent origin of splipalmiviruses and AtenNV1.

Currently, the narnaviruses can be roughly classified into “*Alphanarnavirus*” and “*Betanarnavirus*”. “*Alphanarnavirus*” consists exclusively of non-segmented narnaviruses, encompassing viruses possessing authentic genomes as well as all viruses with rORF. Whereas the “*Betanarnavirus*” accommodates narnaviruses with both non-segmented and segmented genomes (Dinan et al., 2020; Kondo et al., 2022). The recent implementation of NGS has quickly expanded the diversity of narnaviruses, resulting in the proposal of several novel taxa (Chiapello et al., 2020a; Sutela et al., 2020). However, the phylogenetic relationship of this group of viruses remains complex, and further research on taxonomy will be indispensable.

### 4.2 Novel DsRNA viruses with segmented genomes

Two families with dsRNA genomes were characterized in our study, namely *Megabirnaviridae* and *Alternaviridae*. Megabirnaviruses possess bi-segmented dsRNA genomes, with each dsRNA segment containing two ORFs. The dsRNA1 contains ORF1 and 2, which encodes CP and RdRp, respectively. While the dsRNA2 contains two ORFs that encode hypothetical proteins with unknown functions (Sato et al., 2019). Members of the family *Alternaviridae* have a genome consisting of at least three dsRNA segments, each containing only one ORF. The largest dsRNA segment contains an ORF encoding the RdRp (Zhang et al., 2022), and other segments may encode a CP or a hypothetical protein with an unknown function. Previous studies have illustrated that viruses with segmented genomes could originate from non-segmented viruses (Mu et al., 2021; Yang et al., 2021). Given that the majority of dsRNA viruses harbor segmented genomes (Ghabrial et al., 2015; Michalakis and Blanc, 2020), the segmentation of the viral genome should be a preferred scenario for dsRNA virus evolution. In this study, only the dsRNA1 of SlMBV1 and two segments of SlAV1 have been found, whereas other possible dsRNA segments of these viruses remain uncharacterized. As mentioned by others, the determination of segment numbers in mycoviruses could be relatively underestimated since NGS approaches are not efficient in finding non-RdRp segments (Kondo et al., 2022). Consequently, further research will be necessary to search for these additional segments.

### 4.3 A new family of (-) ssRNA viruses discovered

This study identified three lineages of (-) ssRNA viruses, which consisted of the families *Mymonaviridae* and “*Mycoaspiviridae*”, as well as the order *Bunyaviriales*. The *Bunyavirales* is an order of viruses with segmented (-) ssRNA genomes and encompasses a total of 14 families. Three families within this order are known to harbor mycoviruses, namely *Discoviridae*, *Phenuiviridae*, and *Tulasviridae*. Some mycovirus families within this order have been proposed, but have not been approved by ICTV formally yet (Picarelli et al., 2019; Jia et al., 2021; Ruiz-Padilla et al., 2021). During our investigation, we discovered a possible new family within this order. The BLASTp analysis indicated that the members of this family were similar to those viruses in the family *Phasmaviridae*, which typically infect only arthropods (Li et al., 2015). However, the phylogenetic analysis revealed that these phasma-like viruses form a distinct clade that is genetically distant from the *Phasmaviridae* family. This well-defined clade includes both mycoviruses and arthropod viruses, and likely represent a new family in the order *Bunyavirales* (**Figure 9**). Therefore, these viruses should represent a family-level taxon. It is noteworthy that the additional genome segments of segmented viruses belonging to the family “*Mycoaspiviridae*” and the order *Bunyaviriales* have not been characterized in our study. This corresponds with most previous studies (Hamim et al., 2022; Huang et al., 2023) where only the segment that encodes RdRp was detected in segmented (-) ssRNA mycoviruses. Further studies are required to determine whether extra genomic segments exist for these viruses.

### 4.4 The evolutionary implications for cross-species transmission

It has been suggested that the history of virus evolution is a complex interplay driven by both virus-host coevolution and cross-species transmission (CST). The long-term interaction between these two factors has played a crucial role in shaping the current diversity of viruses (Shi et al., 2016; Geoghegan et al., 2017). Mycoviruses, which are usually transmitted intracellularly, are likely to have undergone coevolution with their hosts, indicating a longstanding virus-host interaction since their ancestors (Pearson et al., 2009). This may rationalize why mycovirus infections often result in asymptomatic phenotype (Ghabrial, 1998; Pearson et al., 2009). Nevertheless, another viral evolution pattern, dominated by CST, has become more prominent in the past decade due to the widespread use of NGS technology (Dolja and Koonin, 2018; Wolf et al., 2018). CST is pervasive among different species, and as metagenomics studies have revealed, this phenomenon is a key driver in RNA virus evolution (Wolf et al., 2018). Especially for those eukaryote-infecting viruses in the phylum *Lenarviricota*, whose hosts were originally believed to be limited to fungi and plants (Rastgou et al., 2009; Hillman and Cai, 2013). However, it was subsequently proven that the host range is much broader as metagenomics was employed to study a collection of invertebrates, revealing the pervasive existence of narnaviruses and botourmiavirus in these organisms (Shi et al., 2016). It is noteworthy that invertebrates in nature serve as a reservoir for enormous virus diversity and plays an important role in CST events across different kingdoms (Blanc and Gutierrez, 2015; Dolja and Koonin, 2018; Wolf et al., 2018). Given that fungi are ubiquitous organisms in the environment, the CST between fungi and invertebrates should be universal. Sclerotinia sclerotiorum debilitation-associated DNA virus 1 (SsHADV1) is the most well-studied example of CST between fungi and invertebrates, as it can infect both the fungal host *Sclerotinia sclerotiorum* and the mycophagous fly *Lycoriella ingenua* (Liu et al., 2016). Another case for CST between fungi and invertebrates is the Entomophthovirus of the *Iflaviridae* family, which was identified in *Entomophthora muscae*, a fungal pathogen that infects dipterans. Viruses of the *Iflaviridae* family were previously thought to exclusively infect arthropods, and had never been detected in fungi (Valles et al., 2017; Coyle et al., 2018). In this study, the Hulunbuir Botou tick virus 4, a virus infecting *Dermacentor silvarum*, was found almost identical to SlBV2 (**Table 1**). *D. silvarum* is a tick species with mammalian hosts and distributed in conifer forests of northern China (Liu et al., 2005), largely overlapping with the habitat of *S. luteus*. It can be inferred that *D. silvarum* once formed an intimate ecological association with *S. luteus* on some occasion, rendering SlBV2 an opportunity for host shifting. Such shreds of evidence provide insight into a recent CST event between fungi and arthropods since SlBV2 and Hulunbuir Botou tick virus 4 shared almost identical RdRp sequences. Similarly, a recent tick virome study discovered that botourmiaviruses are pervasive in the tick virome, especially in *D. silvarum* (Ni et al., 2023). These discoveries imply that CST between fungi and ticks is not just an individual case, but has continually happened historically and currently (**Figure 4**). As for those CST that happened within the fungi kingdom, although being frequently reported, the definite mechanism of interspecific transmission remains to be further established (Xie and Jiang, 2014). With the assistance of metagenomics, CST events are found more frequently than we previously believed, and likely act a dominant role in RNA virus evolution (Dolja and Koonin, 2018). It can be predicted that those mycovirus taxa conventionally known to only infect fungi will be found in other organisms in future studies, and vice versa.

In summary, this work is the first study to characterize mycoviruses in *Suillus* species. Nowadays, our overall knowledge of mycoviruses is limited by the biased sampling of host fungi (Myers et al., 2020), which was mainly based on pathogenic fungi and edible mushrooms in the phylum *Ascomycota* and *Basidiomycota*. The mycorrhizal fungi, with a tremendous diversity of associated microorganisms (Shi et al., 2023), can greatly expand the virosphere, thus providing us new insight into the virus evolution. The further implementation of NGS to a broader scale of fungi will inevitably provide us an insight into the full spectrum of mycoviruses, hence providing us a deeper understanding of the viral phylogeny and the intricate relationship of viruses and their hosts.

## Data availability statement

The datasets presented in this study can be found in online repositories. The names of the repository/repositories and accession number(s) can be found in the article/**Supplementary Material**.

## Author contributions

HZL performed the bioinformatics analyses and drafted the manuscript. HPL and PL designed and supervised the research. YFL reviewed and edited the manuscript. YZ, YYL, JX and ZH executed the experiments. All authors contributed to the article and approved the submitted version.
