# Supplementary material for: Virome analysis of an ectomycorrhizal fungus Suillus luteus revealing potential evolutionary implications
Source: Front Cell Infect Microbiol. 2023 Aug 17;13:1229859. doi: 10.3389/fcimb.2023.1229859 (PMC10470027; doi:10.3389/fcimb.2023.1229859)
Supplement: Supplementary file 1 [file DataSheet_1.docx]

Supplementary Material

Virome Analysis of an Ectomycorrhizal Fungus *Suillus luteus* Revealing Potential Evolutionary Implications

Hanzhao Liu, Yifei Zhang, Yingying Liu, Junbo Xiao, Zijie Huang, Yunfeng Li, Huaping Li^*^, Pengfei Li^*^

*** Correspondence:**

Huaping Li: [huaping@scau.edu.cn](mailto:huaping@scau.edu.cn)

Pengfei Li: lipengfei@scau.edu.cn

# Supplementary Figures


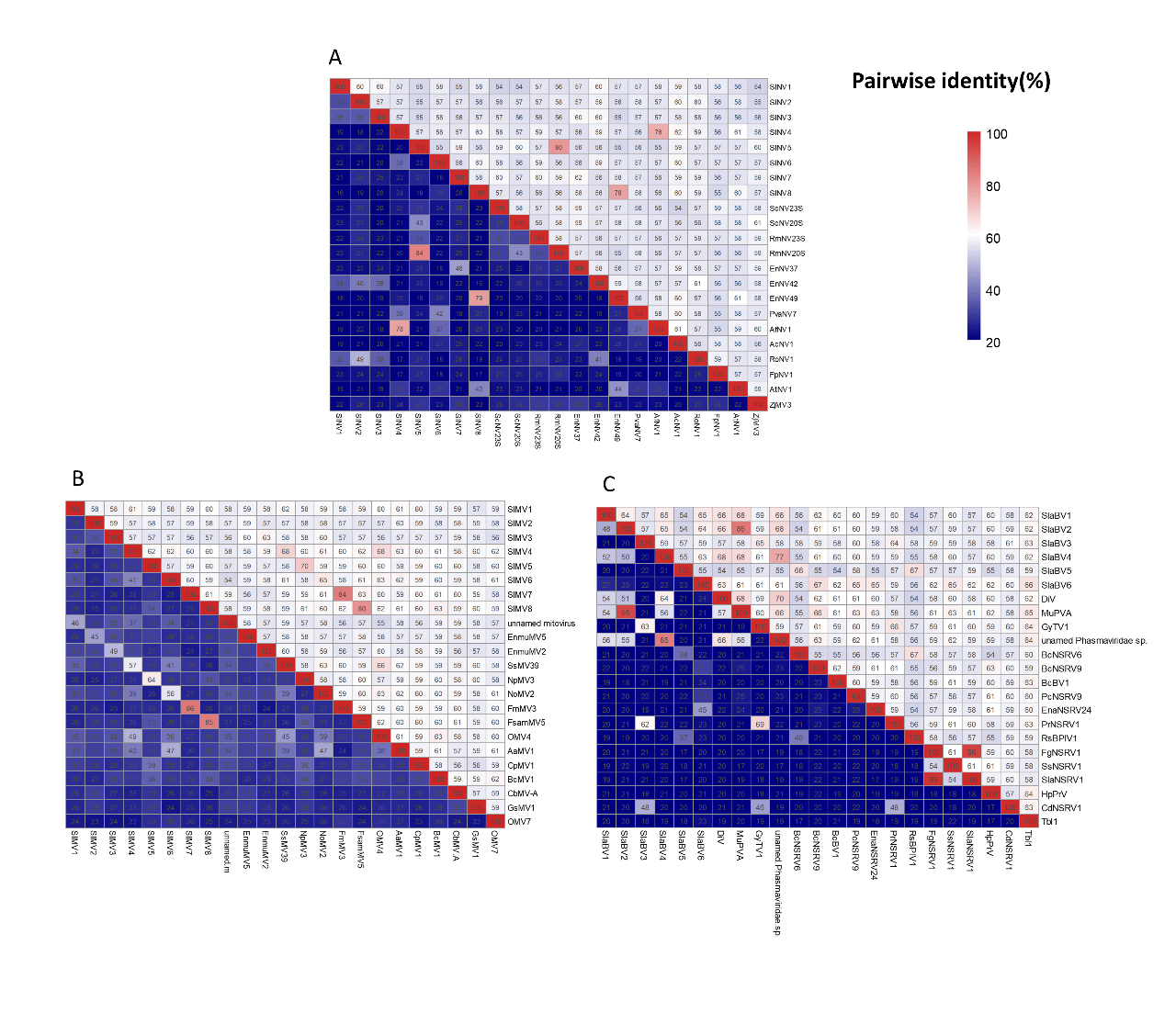


**Figure S1** Pairwise identity comparisons of **(A)** narnaviral sequences, **(B)** mitoviral sequences and **(C)** bunya-related sequences to other known viruses at the amino acid level (lower left) and nucleotide level (upper right). See Table S3 for detailed information on each selected virus used to conduct the analysis.


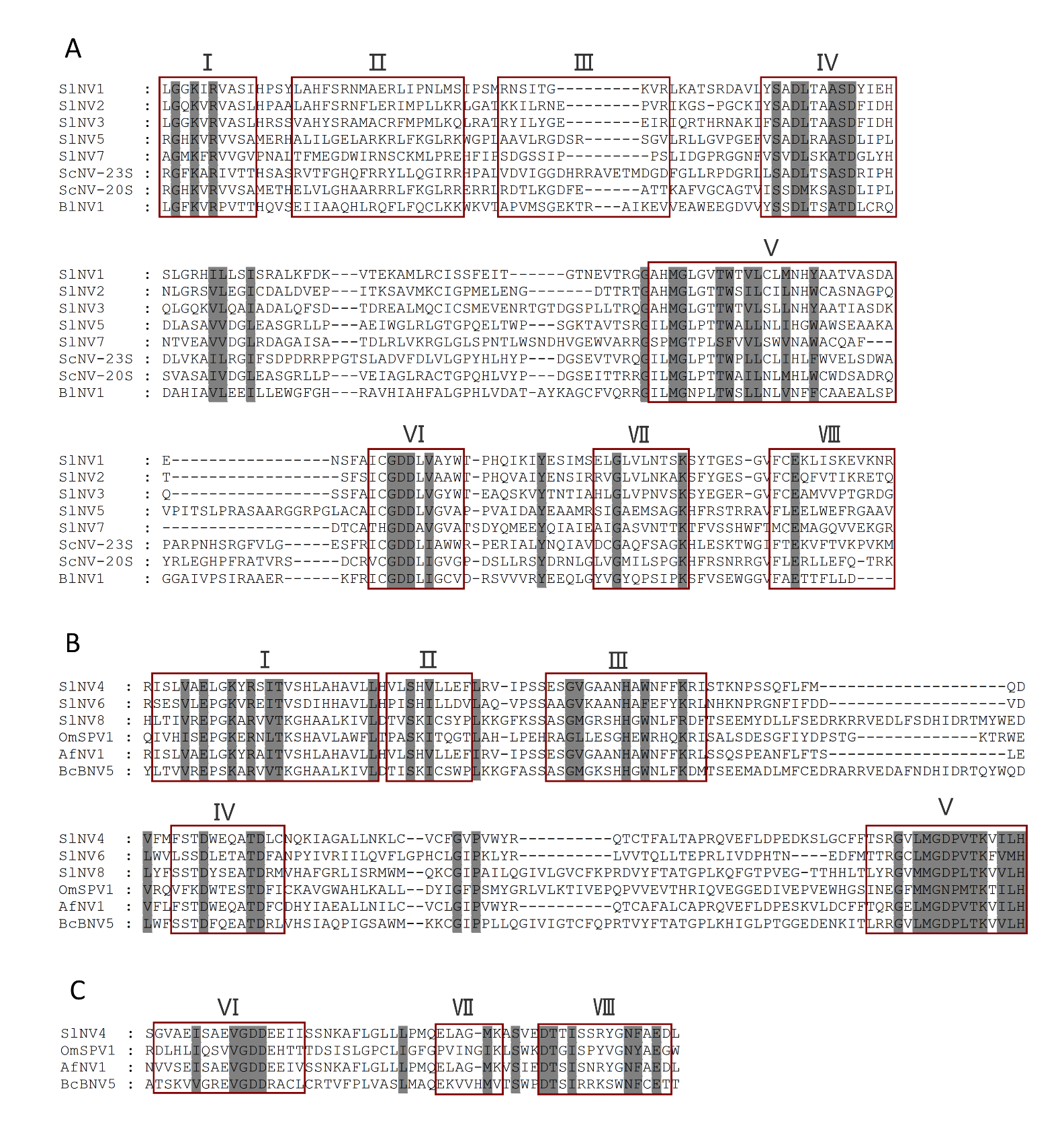


**Figure S2** Multiple alignment of the amino acid sequences of the RdRp encoded by narnaviruses. **(A)** The alignment of alphanarnaviruses conserved motifs of putative RdRp are indicated by the Roman numerals I to Ⅷ. **(B)** The alignment of splipalmiviruses segment 1 conserved motifs of putative RdRp are indicated by the Roman numerals I to V. **(C)** The alignment of splipalmiviruses segment 2 conserved motifs of putative RdRp are indicated by the Roman numerals Ⅵ to Ⅷ. Conserved amino acid residues are marked in grey shading. See Table S4 for detailed information on each selected virus.


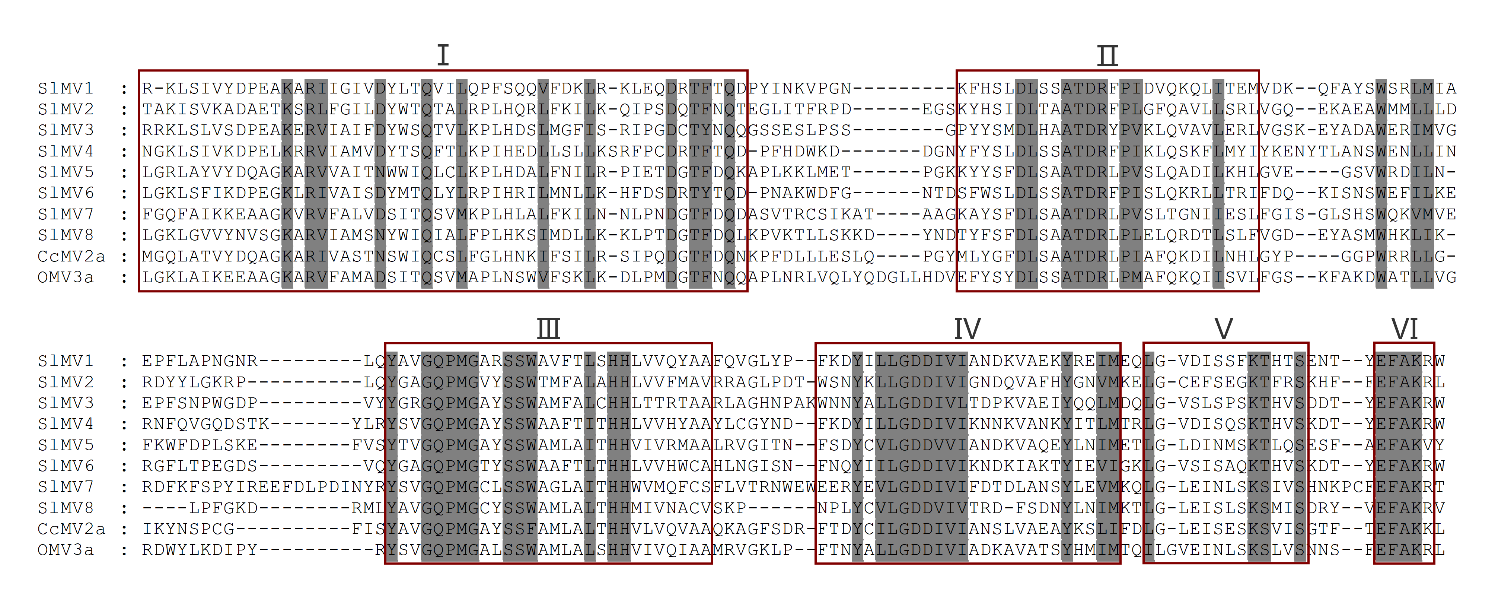


**Figure S3** Multiple alignment of the amino acid sequences of the RdRp encoded by mitoviruses conserved motifs of putative RdRp are indicated by the Roman numerals I to Ⅵ. Conserved amino acid residues are marked in grey shading. See Table S4 for detailed information on each selected virus.


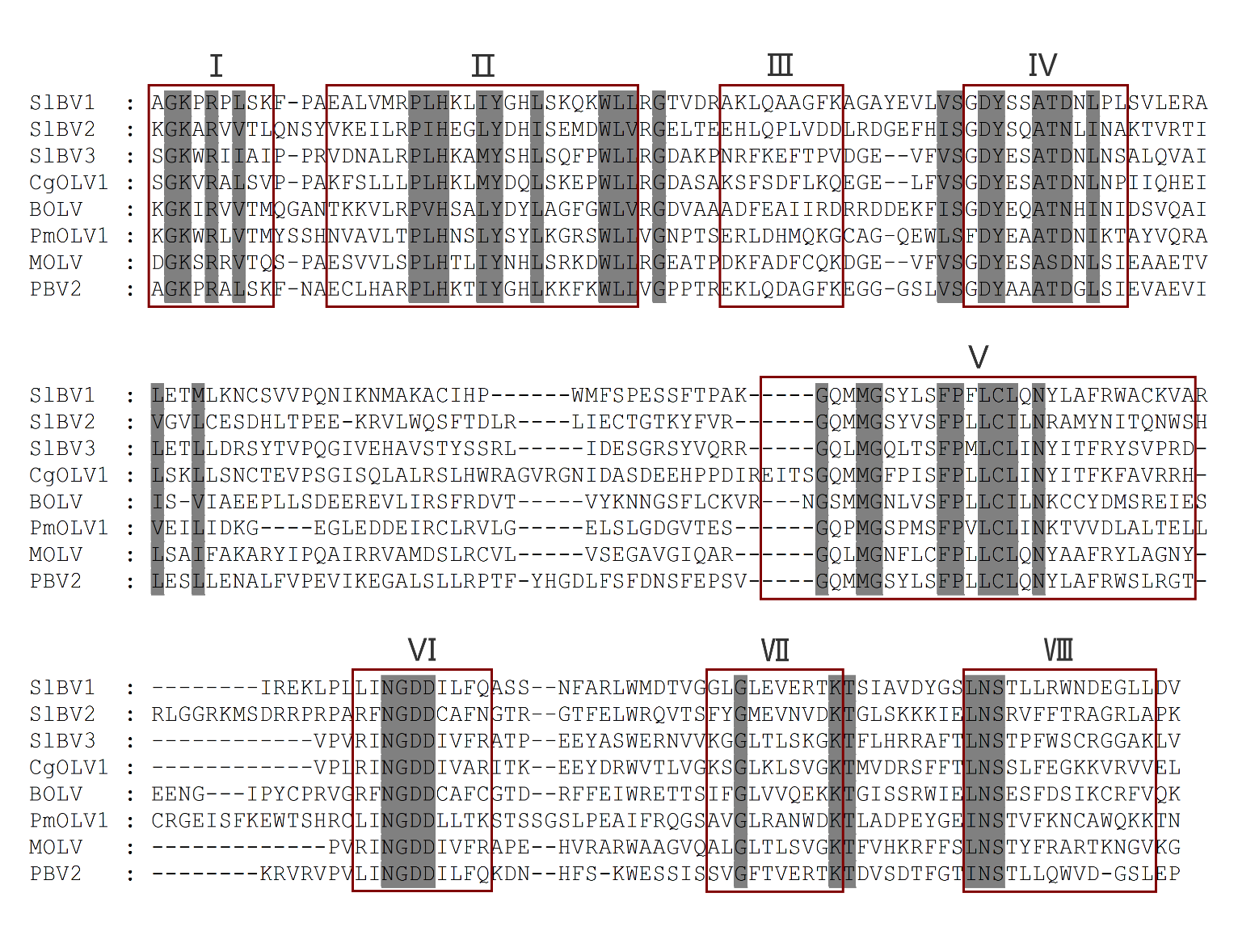


**Figure S4** Multiple alignment of the amino acid sequences of the RdRp encoded by botourmiaviruses conserved motifs of putative RdRp are indicated by the Roman numerals I to Ⅷ. Conserved amino acid residues are marked in grey shading. See Table S4 for detailed information on each selected virus.


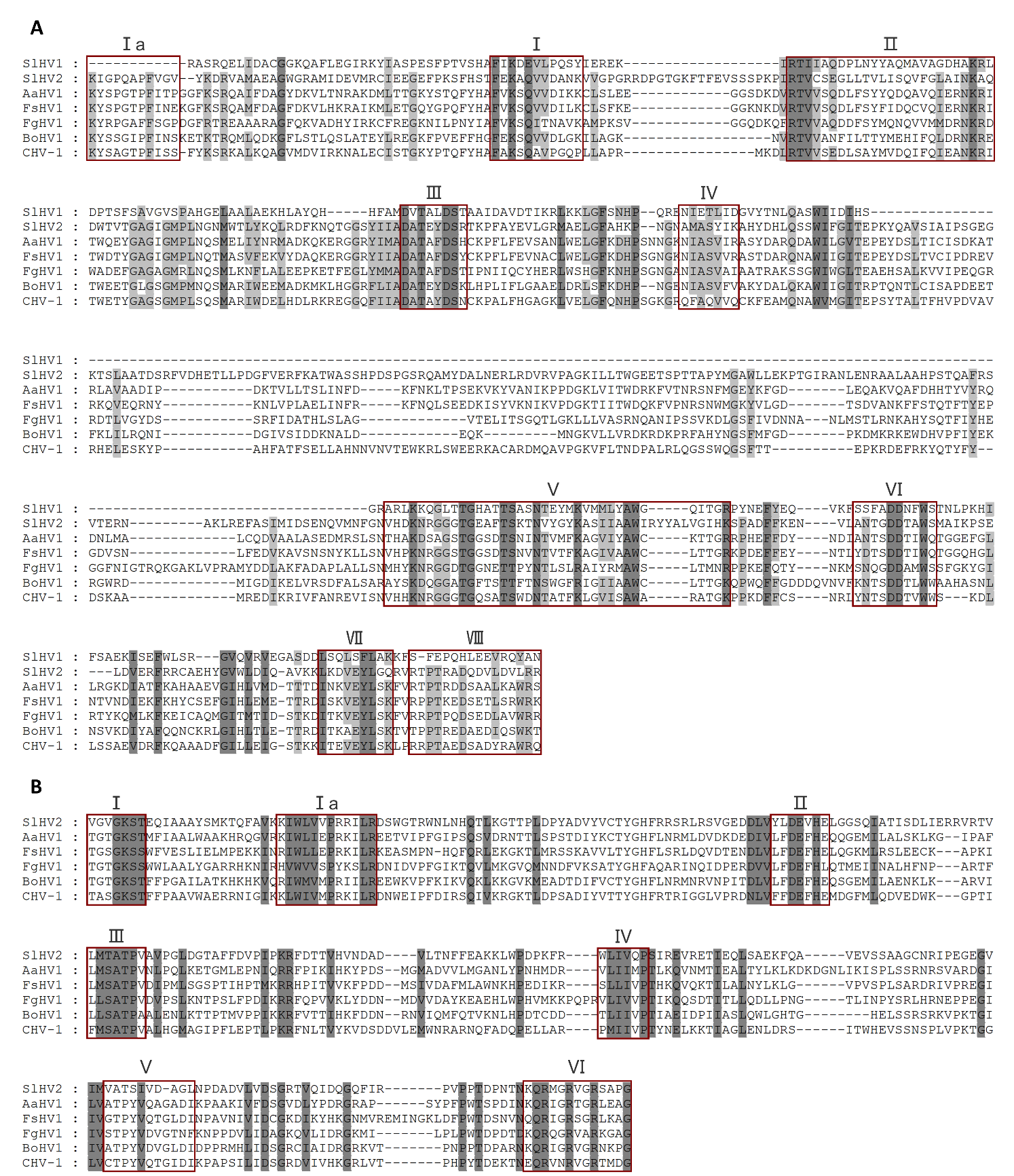


**Figure S5** **(A)** Multiple alignment of the amino acid sequences of the RdRp encoded by hypoviruses. Conserved motifs of putative RdRp are indicated by the Roman numerals Ia to VII. **(B)** Multiple alignment of the amino acid sequences of the helicase domain encoded by hypoviruses. Conserved motifs of putative helicase are indicated by the Roman numerals I to VI. Conserved amino acid residues are marked in grey shading. See Table S4 for detailed information on each selected virus.


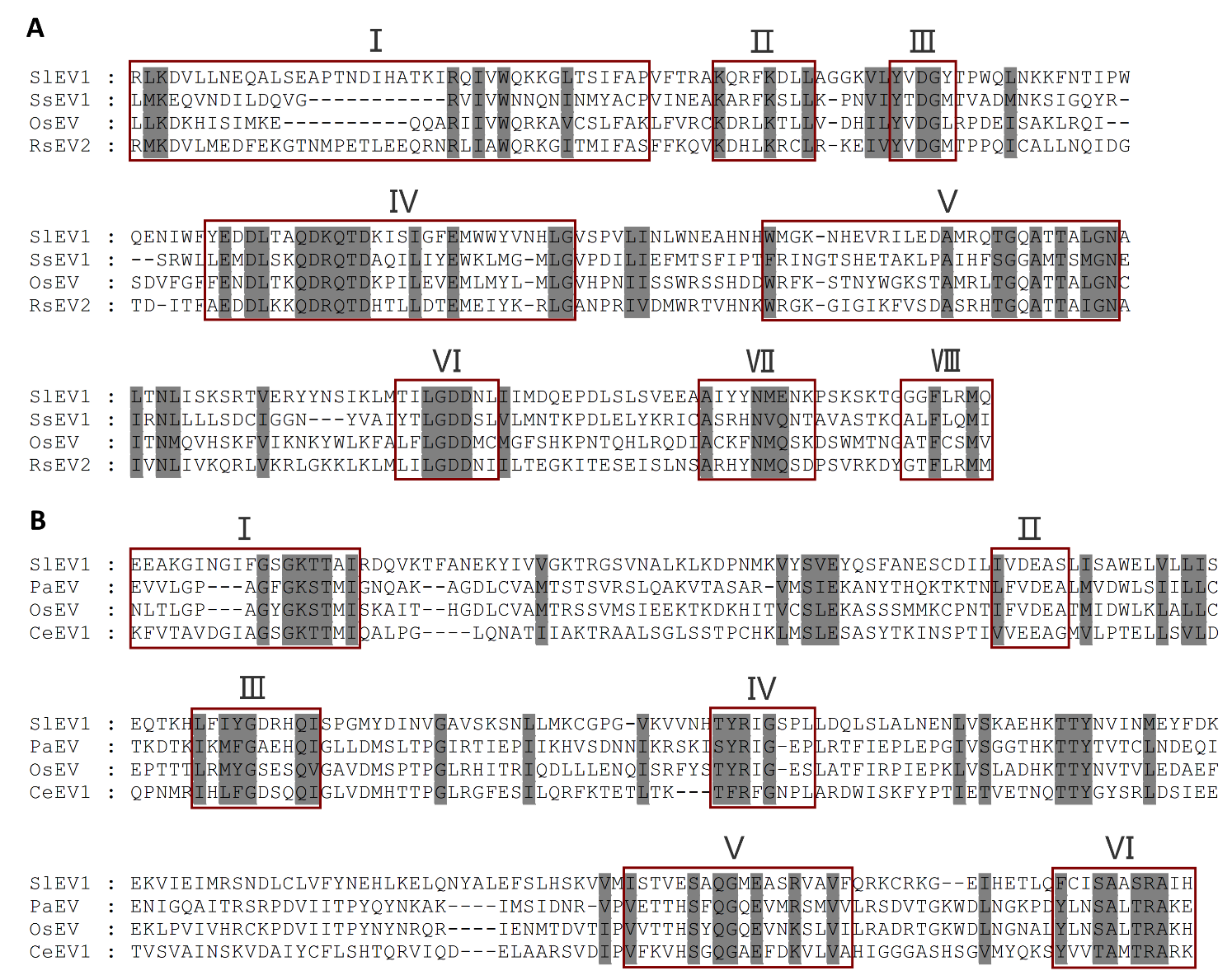


**Figure S6** **(A)** Multiple alignment of the amino acid sequences of the RdRp encoded by endornaviruses. Conserved motifs of putative RdRp are indicated by the Roman numerals I to Ⅷ. **(B)** Multiple alignment of the amino acid sequences of the helicase encoded by endornaviruses. Conserved motifs of putative RdRp are indicated by the Roman numerals I to Ⅵ. Conserved amino acid residues are marked in grey shading. See Table S4 for detailed information on each selected virus.


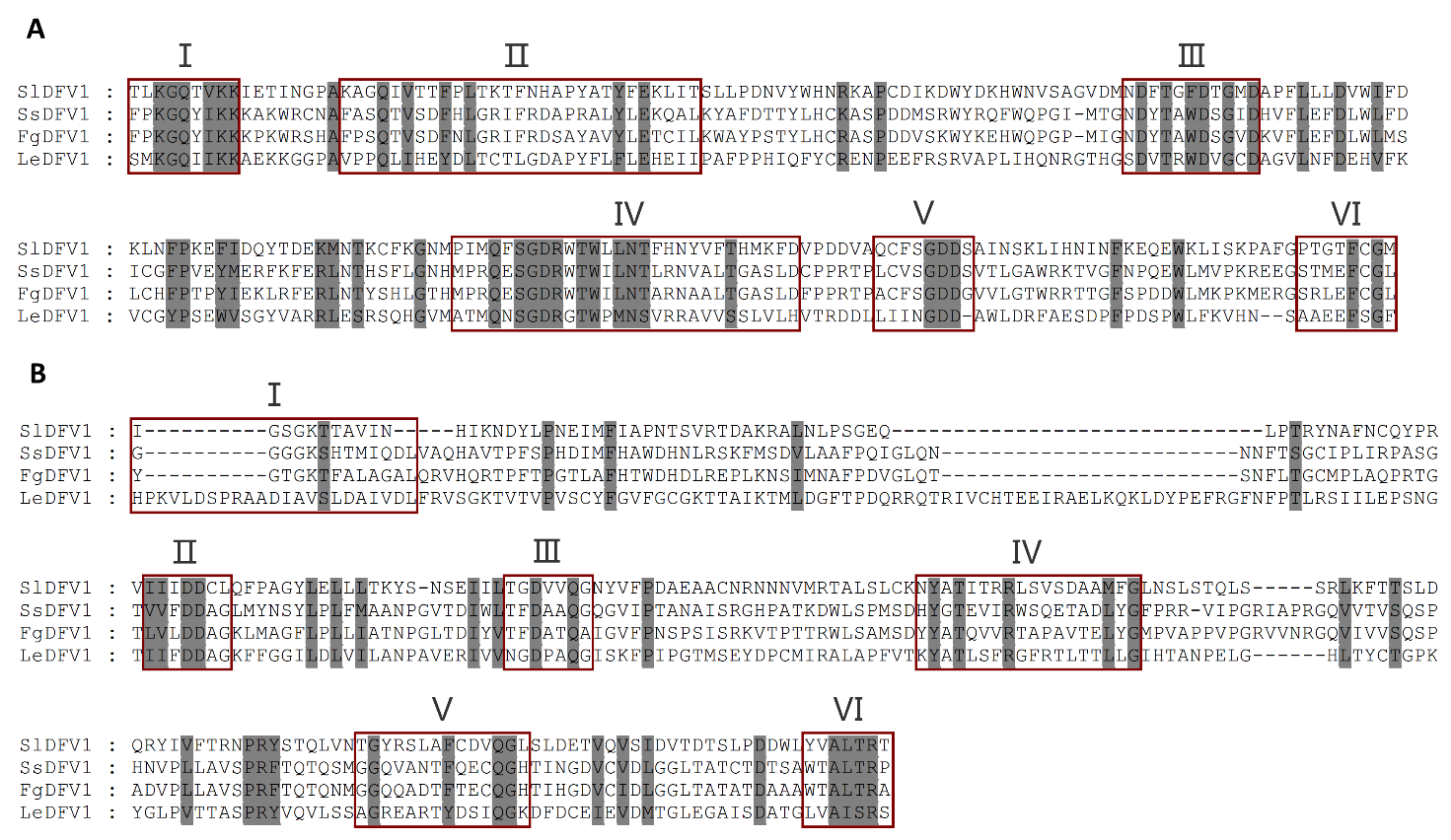


**Figure S7 (A)** Multiple alignment of the amino acid sequences of the RdRp encoded by deltaflexiviruses. Conserved motifs of putative RdRp are indicated by the Roman numerals Ⅰ to Ⅵ. **(B)** Multiple alignment of the amino acid sequences of the helicase encoded by deltaflexiviruses. Conserved motifs of putative RdRp are indicated by the Roman numerals Ⅰ to Ⅵ. Conserved amino acid residues are marked in grey shading. See Table S4 for detailed information on each selected virus.


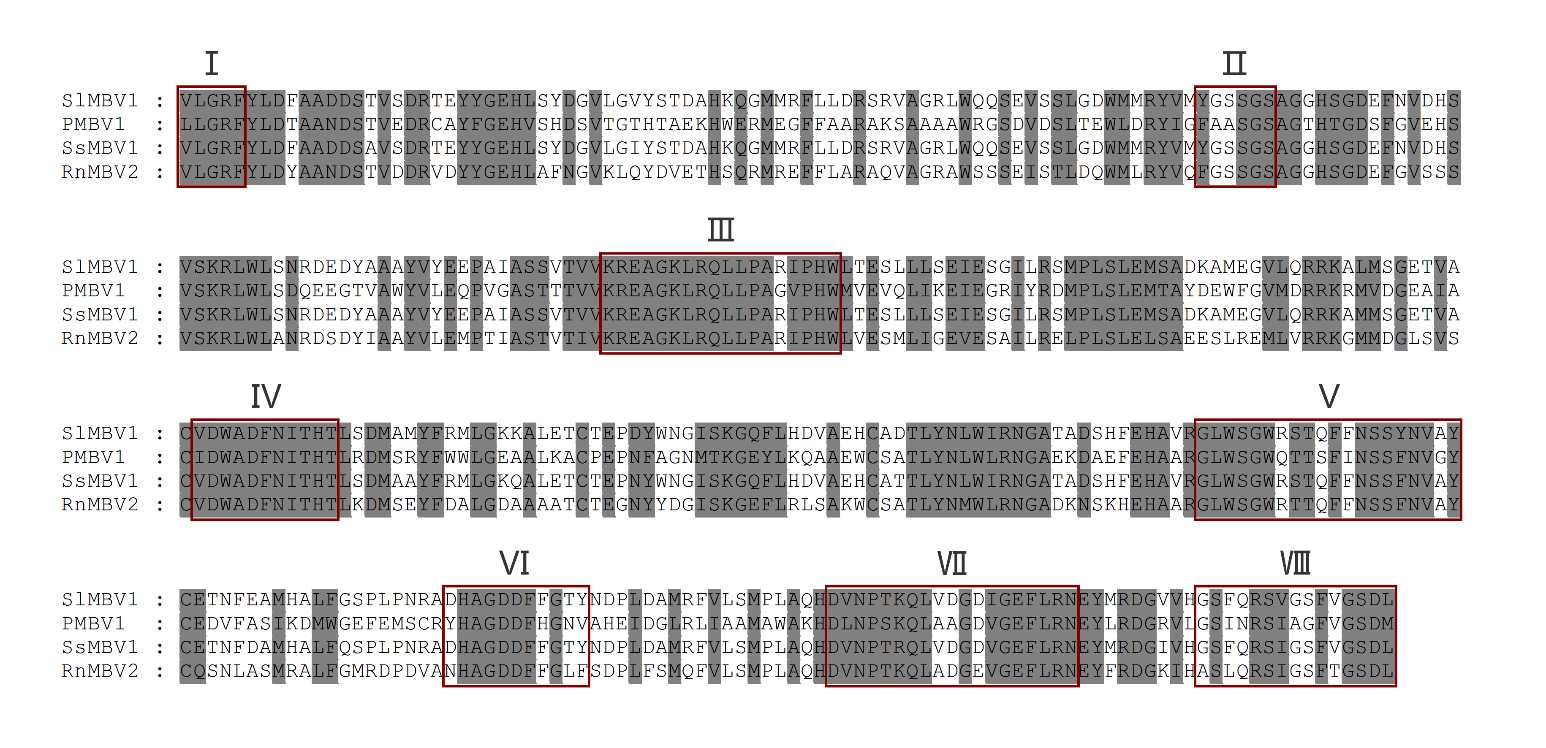


**Figure S8** Multiple alignment of the amino acid sequences of the RdRp encoded by megabirnavirus conserved motifs of putative RdRp are indicated by the Roman numerals I to Ⅷ. Conserved amino acid residues are marked in grey shading. See Table S4 for detailed information on each selected virus.

**
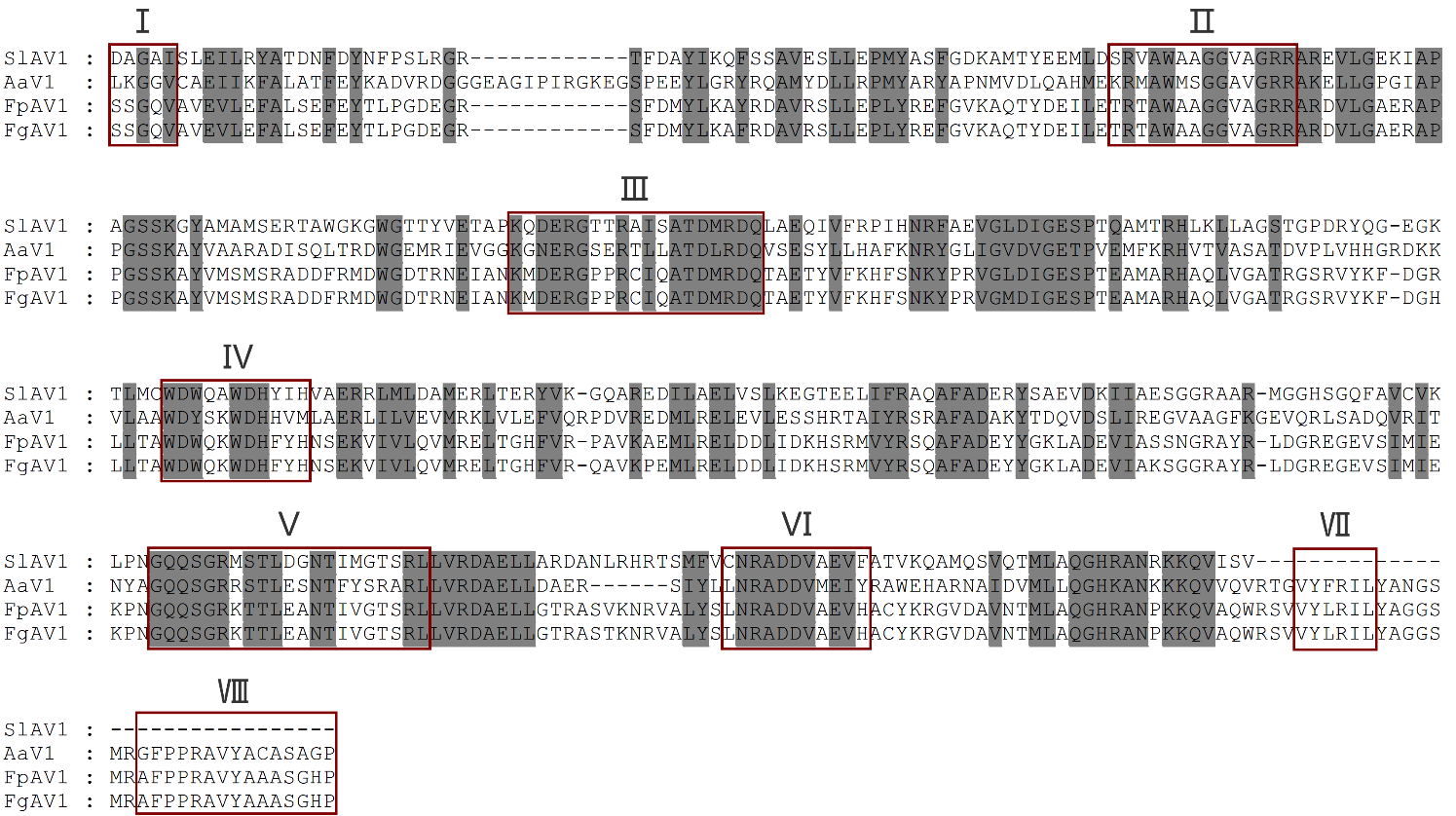
**

**Figure S9** Multiple alignment of the amino acid sequences of the RdRp encoded by alternavirus conserved motifs of putative RdRp are indicated by the Roman numerals I to Ⅷ. Conserved amino acid residues are marked in grey shading. See Table S4 for detailed information on each selected virus.

**
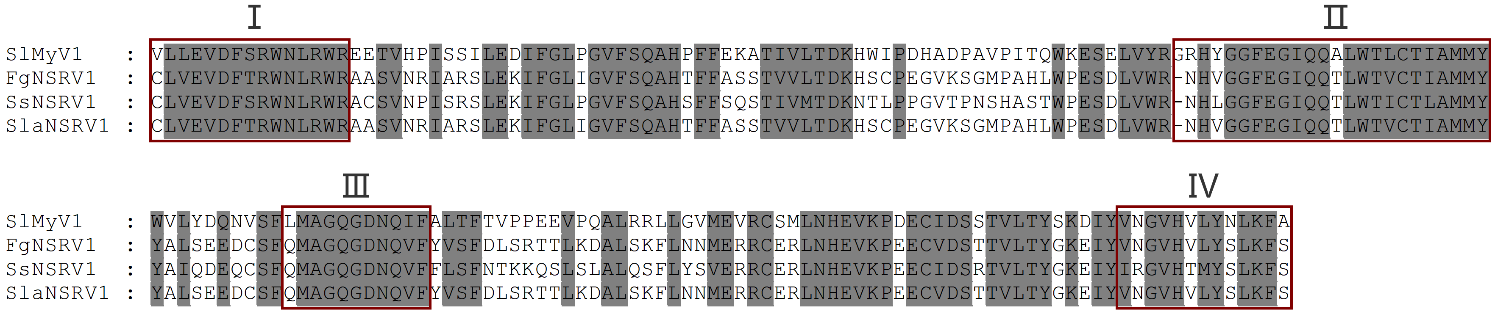
**

**Figure S10** Multiple alignment of the amino acid sequences of the RdRp encoded by mymonavirus conserved motifs of putative RdRp are indicated by the Roman numerals I to IV. Conserved amino acid residues are marked in grey shading. See Table S4 for detailed information on each selected virus.

**
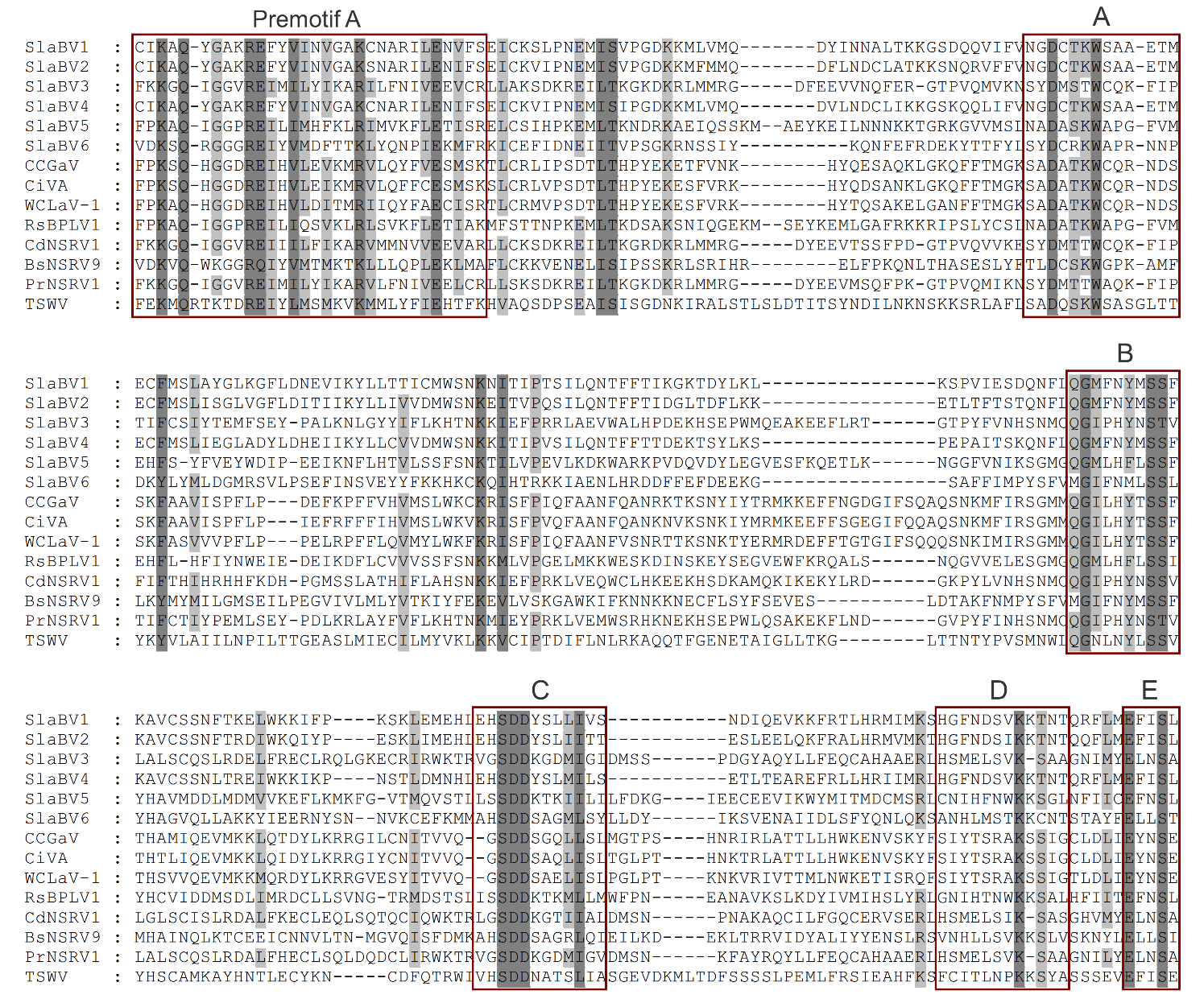
**

**Figure S11** Multiple alignment of the amino acid sequences of the RdRp encoded by mycoviruses within *Bunyavirales* conserved motifs of putative RdRp are indicated by Roman numerals Premotif A and motif A to E. Conserved amino acid residues are marked in grey shading. See Table S4 for detailed information on each selected virus.

**
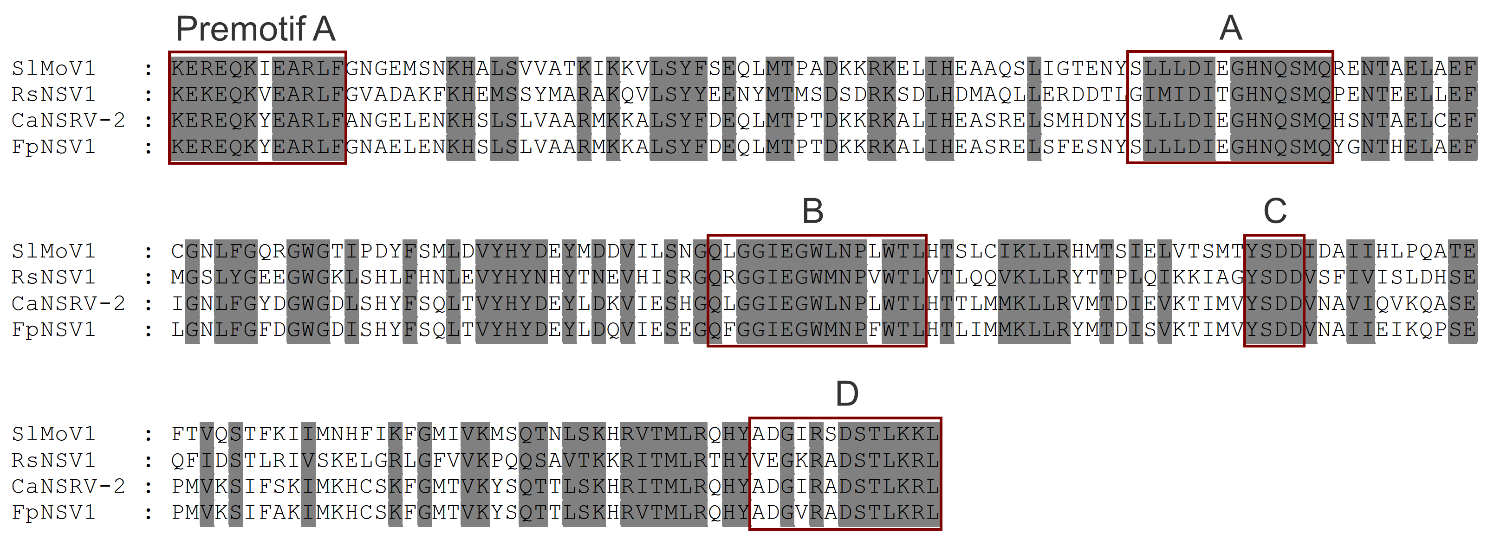
**

**Figure S12** Multiple alignment of the amino acid sequences of the RdRp encoded by mycoophioviruses conserved motifs of putative RdRp are indicated by the Roman numerals Premotif A to D. Conserved amino acid residues are marked in grey shading. See Table S4 for detailed information on each selected virus.

# Supplementary Tables

**Table S1** Viruses used for phylogenetic analyses

| **Virus Name** | **Accession number** | **Family** |
| --- | --- | --- |
| Zhejiang mosquito virus 3 | YP009333331 | *Narnaviridae* |
| Wuhan insect virus 18 | YP009342440 | *Narnaviridae* |
| Rhizopus oryzae narnavirus 1 | BDF97660 | *Narnaviridae* |
| Plasmopara viticola lesion  associated narnavirus 7 | QIR30286 | *Narnaviridae* |
| Saccharomyces 20S RNA narnavirus | NP660178 | *Narnaviridae* |
| Saccharomyces 23S RNA narnavirus | NP660177 | *Narnaviridae* |
| Erysiphe necator associated narnavirus 42 | QJT93774 | *Narnaviridae* |
| Erysiphe necator associated narnavirus 37 | QJT93769 | *Narnaviridae* |
| Erysiphe necator associated narnavirus 49 | QJT93781 | *Narnaviridae* |
| Rhizopus microsporus 20S narnavirus | QBC65280 | *Narnaviridae* |
| Rhizopus microsporus 23S narnavirus | QBC65281 | *Narnaviridae* |
| Aspergillus flavus narnavirus 1 | UAW09567 | *Narnaviridae* |
| Xanthi narna-like virus | MW520409 | *Narnaviridae* |
| Ochlerotatus-associated narna-like virus 1 | KF298275.2 | *Narnaviridae* |
| Ochlerotatus-associated narna-like virus 2 | KF298284.2 | *Narnaviridae* |
| Culex narnavirus 1 | MW226855 | *Narnaviridae* |
| Shahe narna-like virus 4 | KX883556 | *Narnaviridae* |
| Hubei narna-like virus 16 | KX883526 | *Narnaviridae* |
| Point-Douro narna-like virus | MF176258 | *Narnaviridae* |
| Barns Ness breadcrumb sponge  narna-like virus 4 | MF190030 | *Narnaviridae* |
| Wenling narna-like virus 8 | KX883605 | *Narnaviridae* |
| Beihai narna-like virus 24 | KX883500 | *Narnaviridae* |
| Fusarium poae narnavirus 1 | LC150604 | *Narnaviridae* |
| Magnaporthe oryzae narnavirus 2 | BCH36659 | *Narnaviridae* |
| Aspergillus lentulus narnavirus 1 | BCH36643 | *Narnaviridae* |
| Plasmodium vivax Narna-Like virus 1 | QHQ74301 | *Narnaviridae* |
| Wilkie narna-like virus 1 | YP009388589 | *Narnaviridae* |
| Ophiostoma mitovirus 4 | NP_660179 | *Mitoviridae* |
| Leptosphaeria biglobosa mitovirus 1 | UYL94478 | *Mitoviridae* |
| Diaporthe rudis mitovirus 1 | QNS28825 | *Mitoviridae* |
| Fusarium andiyazi mitovirus 1 | QIQ28423 | *Mitoviridae* |
| Alternaria arborescens mitovirus 1 | YP_009270635 | *Mitoviridae* |
| Cronartium ribicola mitovirus 1 | YP_009259369 | *Mitoviridae* |
| Puccinia striiformis mitovirus 1 | AZY91986 | *Mitoviridae* |
| Agaricus bisporus mitovirus 1 | AQM32767 | *Mitoviridae* |
| Hymenoscyphus fraxineus mitovirus 1 | AZG04326 | *Mitoviridae* |
| Gigaspora margarita mitovirus 1 | YP_009553175 | *Mitoviridae* |
| Tuber aestivum mitovirus | YP_004564622 | *Mitoviridae* |
| Heterobasidion mitovirus 1 | AIF33766.2 | *Mitoviridae* |
| Epicoccum nigrum mitovirus 1 | QDB74989 | *Mitoviridae* |
| Neofusicoccum parvum mitovirus 1 | QDB74992 | *Mitoviridae* |
| Cryphonectria parasitica mitovirus 1 | L31849 | *Mitoviridae* |
| Botrytis cinerea mitovirus 1 | QLF49185 | *Mitoviridae* |
| Sclerotinia sclerotiorum mitovirus 3 | YP_009182164 | *Mitoviridae* |
| Ophiostoma mitovirus 3a | NP_660176 | *Mitoviridae* |
| Sclerotinia homoeocarpa mitovirus | AAO21337 | *Mitoviridae* |
| Ceratobasidium mitovirus A | AOX47577 | *Mitoviridae* |
| Rhizoctonia oryzae-sativae mitovirus 1 | YP_009249807 | *Mitoviridae* |
| Geopora sumneriana mitovirus 1 | MN043682 | *Mitoviridae* |
| Ophiostoma mitovirus 7 | AGT55877 | *Mitoviridae* |
| Entomophthora muscae mitovirus 5 | DAC76944 | *Mitoviridae* |
| Entomophthora muscae mitovirus 2 | DAC76941 | *Mitoviridae* |
| Sclerotinia sclerotiorum mitovirus 39 | QUE49178 | *Mitoviridae* |
| Neofusicoccum parvum mitovirus 3 | QTE76052 | *Mitoviridae* |
| Nigrospora oryzae mitovirus 2 | AZP53929 | *Mitoviridae* |
| Fusarium mangiferae mitovirus 3 | UBZ25882 | *Mitoviridae* |
| Fusarium sambucinum mitovirus 5 | BCP96876 | *Mitoviridae* |
| Mitovirus sp. | QDH89715 | *Mitoviridae* |
| Cladosporium uredinicola ourmiavirus 1 | QDB75001 | *Botourmiaviridae* |
| Oidiodendron maius ourmia-like virus 1 | MN736966 | *Botourmiaviridae* |
| Soybean leaf-associated ourmiavirus 1 | NC_043521 | *Botourmiaviridae* |
| Sclerotinia sclerotiorum  ourmia-like virus 1 | NC_055144 | *Botourmiaviridae* |
| Plasmopara viticola lesion  associated ourmia-like virus 44 | QGY72574 | *Botourmiaviridae* |
| Plasmopara viticola lesion  associated ourmia-like virus 69 | QGY72599 | *Botourmiaviridae* |
| Plasmopara viticola lesion  associated ourmia-like virus 63 | WAK77949 | *Botourmiaviridae* |
| Plasmopara viticola lesion  associated ourmia-like virus 45 | QGY72575 | *Botourmiaviridae* |
| Plasmopara viticola lesion  associated ourmia-like virus 20 | QGY72550 | *Botourmiaviridae* |
| Plasmopara viticola lesion  associated ourmia-like virus 25 | QGY72555 | *Botourmiaviridae* |
| Phaeoacremonium minimum  ourmia-like virus 2 | QDB75007 | *Botourmiaviridae* |
| Cladosporium cladosporioides  ourmia-like virus 1 | QDB74999 | *Botourmiaviridae* |
| Colletotrichum gloeosporioides  ourmia-like virus 1 | QDW80875 | *Botourmiaviridae* |
| Magnaporthe oryzae ourmia-like virus 1 | QDW80874 | *Botourmiaviridae* |
| Botrytis cinerea ourmia-like virus 1 | QJT73667 | *Botourmiaviridae* |
| Pyricularia oryzae ourmia-like virus 1 | BBF90576 | *Botourmiaviridae* |
| Plasmopara viticola lesion associated  ourmia-like virus 1 | QGY72531 | *Botourmiaviridae* |
| Phaeoacremonium minimum  ourmia-like virus 3 | WAK77887 | *Botourmiaviridae* |
| Botrytis cinerea ourmia-like virus 4 | QJT73670 | *Botourmiaviridae* |
| Botrytis ourmia-like virus | QLF49182 | *Botourmiaviridae* |
| Plasmopara viticola lesion  associated ourmia-like virus 81 | QGY72611 | *Botourmiaviridae* |
| Epicoccum nigrum ourmia-like virus 1 | QDB75003 | *Botourmiaviridae* |
| Sclerotinia sclerotiorum  ourmia-like virus 2 | YP_010084712 | *Botourmiaviridae* |
| Aspergillus pseudoviridinutans  botourmiavirus 1 | BCH36653 | *Botourmiaviridae* |
| Phoma matteucciicola  ourmia-like virus 1 | QIP68359 | *Botourmiaviridae* |
| Pestalotiopsis botourmiavirus 2 | QTH80196 | *Botourmiaviridae* |
| Botourmiaviridae sp. | WAK77894 | *Botourmiaviridae* |
| Tonghua Botou tick virus 3 | UYL95452 | *Botourmiaviridae* |
| Hulunbuir Botou tick virus 4 | UYL95442 | *Botourmiaviridae* |
| Tianjin Botou tick virus 3 | UYL95449 | *Botourmiaviridae* |
| Luoyang Botou tick virus 1 | UYL95433 | *Botourmiaviridae* |
| Luoyang Botou tick virus 2 | UYL95453 | *Botourmiaviridae* |
| Lianyungang Botou tick virus 1 | UYL95429 | *Botourmiaviridae* |
| Lianyungang Botou tick virus 3 | UYL95437 | *Botourmiaviridae* |
| Cryphonectria hypovirus 1 | M57938 | *Hypoviridae* |
| Cryphonectria hypovirus 2 | NC_003534 | *Hypoviridae* |
| Cryphonectria hypovirus 3 | NC_000960 | *Hypoviridae* |
| Fusarium sacchari hypovirus 1 | MN295969 | *Hypoviridae* |
| Botrytis cinerea hypovirus 1 | NC_037659 | *Hypoviridae* |
| Sclerotinia sclerotiorum hypovirus 3 | MF444222 | *Hypoviridae* |
| Sclerotinia sclerotiorum hypovirus 1 | NC_015939 | *Hypoviridae* |
| Bipolaris oryzae hypovirus 1 | MH316122 | *Hypoviridae* |
| Beihai hypo-like virus 1 | KX883006 | *Hypoviridae* |
| Beihai sipunculid worm virus 6 | KX883005 | *Hypoviridae* |
| Fusarium graminearum hypovirus 2 | KP208178 | *Hypoviridae* |
| Fusarium poae hypovirus 1 | LC150612 | *Hypoviridae* |
| Sclerotium rolfsii hypovirus 2 | MH766497 | *Hypoviridae* |
| Sclerotium rolfsii hypovirus 3 | MH766498 | *Hypoviridae* |
| Sclerotium rolfsii hypovirus 4 | MH766499 | *Hypoviridae* |
| Sclerotium rolfsii hypovirus 8 | MH766503 | *Hypoviridae* |
| Botrytis cinerea hypovirus 4 | MN617171 | *Hypoviridae* |
| Rhizoctonia solani hypovirus 1 | MK558259 | *Hypoviridae* |
| Sclerotinia sclerotiorum hypovirus 6 | MH766501 | *Hypoviridae* |
| Alternaria dianthicola hypovirus 1 | UYZ32447 | *Hypoviridae* |
| Apis hypovirus 2 | UCR92524 | *Hypoviridae* |
| Alternaria alternata hypovirus 1 | QFR36339 | *Hypoviridae* |
| Fusarium graminearum hypovirus 1 | AGC75065 | *Hypoviridae* |
| Sclerotinia sclerotiorum endornavirus 1 | YP_008169851 | *Endornaviridae* |
| Chalara elegans endornavirus 1 | ADN43901 | *Endornaviridae* |
| Oryza sativa endornavirus | D32136 | *Endornaviridae* |
| Rhizopus microspores endornavirus 1 | BDF97664 | *Endornaviridae* |
| Rhizoctonia solani endornavirus 2 | YP_010086587 | *Endornaviridae* |
| Tuber aestivum endornavirus | HQ380014 | *Endornaviridae* |
| Potato virus X | NC_011620 | *Alphaflexiviridae* |
| Indian citrus ringspot virus | NP_203553 | *Alphaflexiviridae* |
| Botrytis virus X | NP_932306 | *Alphaflexiviridae* |
| Sclerotinia sclerotiorum  debilitation-associated RNA virus | NC_007415 | *Alphaflexiviridae* |
| Shallot virus X | NP_620648 | *Alphaflexiviridae* |
| Garlic virus A | NC_003375 | *Alphaflexiviridae* |
| Apple stem pitting virus | AEP02955 | *Betaflexiviridae* |
| Apricot vein clearing associated virus | AKN09002 | *Betaflexiviridae* |
| Cherry necrotic rusty mottle virus | BAU25805 | *Betaflexiviridae* |
| Cherry rusty mottle associated virus | AKN20442 | *Betaflexiviridae* |
| Banana mild mosaic virus | NC_002729 | *Betaflexiviridae* |
| Grapevine virus A | NC_003604 | *Betaflexiviridae* |
| Botrytis virus F | NC_002604 | *Gammaflexiviridae* |
| Entoleuca gammaflexivirus 2 | MF375884 | *Gammaflexiviridae* |
| Turnip yellow mosaic virus | NC_004063 | *Tymoviridae* |
| Andean potato latent virus | NC_020470 | *Tymoviridae* |
| Grapevine Syrah Virus-1 | NC_012484 | *Tymoviridae* |
| Maize rayado fino virus | NC_002786 | *Tymoviridae* |
| Citrus sudden death-associated virus | NC_006950 | *Tymoviridae* |
| Sclerotinia sclerotiorum deltaflexivirus 2 | NC_040649 | *Deltaflexiviridae* |
| Fusarium graminearum deltaflexivirus 1 | NC_030654 | *Deltaflexiviridae* |
| Sclerotinia sclerotiorum deltaflexivirus 1 | NC_038977 | *Deltaflexiviridae* |
| Lentinula edodes deltaflexivirus 1 | MN744723 | *Deltaflexiviridae* |
| Agaricus bisporus virus 7 | KY357491 | *Deltaflexiviridae* |
| Rhizoctonia solani flexivirus 1 | YP_009268715 | *Deltaflexiviridae* |
| Sclerotinia sclerotiorum megabirnavirus 1 | KP686398 | *Megabirnaviridae* |
| Rosellinia necatrix megabirnavirus 1 | AB512282 | *Megabirnaviridae* |
| Rosellinia necatrix megabirnavirus 2 | LC062704 | *Megabirnaviridae* |
| Rosellinia necatrix megabirnavirus 3 | LC333756 | *Megabirnaviridae* |
| Pleosporales megabirnavirus 1 | KT601119 | *Megabirnaviridae* |
| Entoleuca megabirnavirus 1 | MF375886 | *Megabirnaviridae* |
| Phlebiopsis gigantea mycovirus | YP003541123 | *Megabirnaviridae* |
| Alternaria alternata virus 1 | YP001976142 | *Alternaviridae* |
| Fusarium incarnatum alternavirus 1 | AYJ09265 | *Alternaviridae* |
| Fusarium poae alternavirus 1 | YP009272952 | *Alternaviridae* |
| Aspergillus foetidus dsRNA mycovirus | YP007353985 | *Alternaviridae* |
| Fusarium graminearum alternavirus 1 | YP009449439 | *Alternaviridae* |
| Fusarium nanum alternavirus 1 | OK318880 | *Alternaviridae* |
| Cordyceps chanhua alternavirus 1 | OK481552 | *Alternaviridae* |
| Diaporthe alternavirus 1 | BDQ13829 | *Alternaviridae* |
| Colletotrichum gloeosporioides chrysovirus 1 | KT581957 | *Chrysoviridae* |
| Cryphonectria nitschkei chrysovirus 1 | GQ290649.2 | *Chrysoviridae* |
| Macrophomina phaseolina chrysovirus 1 | KP900886 | *Chrysoviridae* |
| Penicillium chrysogenum virus | AF296439 | *Chrysoviridae* |
| Helminthosporium victoriae 145S virus | YP052858 | *Chrysoviridae* |
| Puccinia striiformis totivirus 2 | ATO91009 | *Totiviridae* |
| Red clover powdery mildew-associated totivirus 5 | YP009182188 | *Totiviridae* |
| Red clover powdery mildew-associated totivirus 1 | BAT62476 | *Totiviridae* |
| Black raspberry virus F | YP001497151 | *Totiviridae* |
| Trichoderma koningiopsis totivirus 1 | QGA70771 | *Totiviridae* |
| Umbelopsis ramanniana virus 4 | VUD77425 | *Totiviridae* |
| Sclerotinia sclerotiorum  negative-stranded RNA virus 1 | YP_009094317 | *Mymonaviridae* |
| Sclerotinia sclerotiorum  negative-stranded RNA virus 1-A | AWY11008 | *Mymonaviridae* |
| Sclerotinia sclerotiorum  negative-stranded RNA virus 2 | ALD89145 | *Mymonaviridae* |
| Sclerotinia sclerotiorum  negative-stranded RNA virus 3 | YP_009129259 | *Mymonaviridae* |
| Sclerotinia sclerotiorum  negative-stranded RNA virus 3-A | AWY11025 | *Mymonaviridae* |
| Sclerotinia sclerotiorum  negative-stranded RNA virus 4 | YP_009666274 | *Mymonaviridae* |
| Sclerotinia sclerotiorum  negative-stranded RNA virus 7 | AWY11040 | *Mymonaviridae* |
| Plasmopara viticola lesion  associated mymonavirus 1 | QHD64779 | *Mymonaviridae* |
| Plasmopara viticola lesion  associated mononega virus 2 | QHD64785 | *Mymonaviridae* |
| Fusarium graminearum  negative-stranded RNA virus 1 | ATP75709 | *Mymonaviridae* |
| Alternaria tenuissima  negative-stranded RNA virus 1 | QDB75013 | *Mymonaviridae* |
| Botrytis cinerea mymonavirus 1 | AXS76906 | *Mymonaviridae* |
| Soybean leaf-associated  negative-stranded RNA virus 1 | ALM62220 | *Mymonaviridae* |
| Soybean leaf-associated  negative-stranded RNA virus 2 | ALM62227 | *Mymonaviridae* |
| Soybean leaf-associated  negative-stranded RNA virus 3 | ALM62228 | *Mymonaviridae* |
| Soybean leaf-associated  negative-stranded RNA virus 4 | ALM62229 | *Mymonaviridae* |
| Penicillium adametzioides  negative-stranded RNA virus 1 | QDB75019 | *Mymonaviridae* |
| Penicillium glabrum  negative-stranded RNA virus 1 | QDB75014 | *Mymonaviridae* |
| Plasmopara viticola lesion  associated mononegaambi virus 1 | QHD64768 | *Mymonaviridae* |
| Plasmopara viticola lesion  associated mononegaambi virus 2 | QHD64771 | *Mymonaviridae* |
| Plasmopara viticola lesion  associated mononegaambi virus 3 | QHD64773 | *Mymonaviridae* |
| Plasmopara viticola lesion  associated mononegaambi virus 4 | QHD64774 | *Mymonaviridae* |
| Plasmopara viticola lesion  associated mononegaambi virus 5 | QHD64776 | *Mymonaviridae* |
| Plasmopara viticola lesion  associated mononegaambi virus 6 | QHD64777 | *Mymonaviridae* |
| Gysinge virus | QGA70913 | *Mymonaviridae* |
| Auricularia heimuer  negative-stranded RNA virus 1 | QJP04103 | *Mymonaviridae* |
| Xinjiang mymona-like virus 2 | QYF49867 | *Mymonaviridae* |
| Aspergillus fumigatus  negative-stranded RNA virus 1 | BCH36617 | *Discoviridae* |
| Penicillium discovirus | MF142458 | *Discoviridae* |
| Penicillium roseopurpureum  negative ssRNA virus 1 | AYP71800 | *Discoviridae* |
| Coniothyrium diplodiella  negative-stranded RNA virus 1 | QDB75015 | *Discoviridae* |
| Plasmopara viticola lesion associated mycobunyavirales-like virus 4 | QGY72641 | *Discoviridae* |
| Plasmopara viticola lesion associated mycobunyavirales-like virus 8 | MN585278 | *Discoviridae* |
| Plasmopara viticola lesion associated  mycobunyavirales-like virus 9 | QJX19792 | *Discoviridae* |
| Rhizoctonia solani  bunya/phlebo-like virus 1 | MK507779 | *"Mycophleboviridae"* |
| Botrytis cinerea negative-stranded RNA virus 6 | QJT73694 | *"Mycophleboviridae"* |
| Citrus concave gum-associated virus | KX960112 | *Phenuiviridae* |
| Citrus virus A | QEU56214 | *Phenuiviridae* |
| Botrytis cinerea bocivirus 1 | MN617081 | *Phenuiviridae* |
| Watermelon crinkle leaf-associated virus 1 | ASY01340 | *Phenuiviridae* |
| Botrytis cinerea  negative stranded RNA virus 9 | MT157409 | *"Mybuviridae"* |
| Botrytis cinerea  negative stranded RNA virus 10 | MT157410 | *"Mybuviridae"* |
| Beihai sesarmid crab virus 5 | APG79283 | *"Mybuviridae"* |
| Phytophthora condilina  negative stranded RNA virus 9 | QTT61001 | *"Mybuviridae"* |
| Halophytophthora RNA virus 1 | QLF99168 | *"Mybuviridae"* |
| Macrophomina phaseolina mycobunyavirus 3 | QOE55581 | *"Mybuviridae"* |
| Erysiphe necator associated  negative-stranded RNA virus 24 | QJW70356 | *"Mycobunyaviridae"* |
| Macrophomina phaseolina mycobunyavirus 1 | QOE55579 | *"Mycobunyaviridae"* |
| Macrophomina phaseolina mycobunyavirus 2 | QOE55580 | *"Mycobunyaviridae"* |
| Macrophomina phaseolina mycobunyavirus 4 | QOE55582 | *"Mycobunyaviridae"* |
| Botrytis cinerea negative-stranded RNA virus 1 | YP_009182153 | *"Mycobunyaviridae"* |
| Hemipteran phasma-related virus | QMP82378 | *Phasmaviridae* |
| Hymenopteran phasma-related virus | WBM84623 | *Phasmaviridae* |
| Ferak virus | AKN56888 | *Phasmaviridae* |
| Sanxia Water Strider Virus 2 | KM817675 | *Phasmaviridae* |
| Jonchet virus | AKN56871 | *Phasmaviridae* |
| Niukluk phantom virus | MN168168 | *Phasmaviridae* |
| Largemouth bass bunyavirus | MN119734 | *Peribunyaviridae* |
| Herbert virus | JQ659256 | *Peribunyaviridae* |
| Kibale virus | KF590577 | *Peribunyaviridae* |
| Lakamha virus | MN092355 | *Peribunyaviridae* |
| Chilibre virus | MK330762 | *Peribunyaviridae* |
| Potosi virus | MF066368 | *Peribunyaviridae* |
| Northway virus | MH484312 | *Peribunyaviridae* |
| Guyuan tick virus 1 | UYL95512 | *Peribunyaviridae* |
| Ixodes scapularis associated virus 3 | AUW34410 | possible new family |
| Ditton virus | AWA82278 | possible new family |
| Mucor phasmavirus A | QED42998 | possible new family |
| Phasmaviridae sp. | WAK75648 | possible new family |
| Mucorales RNA virus 1 | AMK47917 | possible new family |
| Yunnan bunya-like virus | QYF49510 | possible new family |
| Cladosporium cladosporioides  negative-stranded RNA virus 1 | QDB75017 | *"Mycoaspiviridae"* |
| Fusarium poae negative-stranded virus 1 | YP009272911 | *"Mycoaspiviridae"* |
| Sclerotinia sclerotiorum  negative-stranded RNA virus 9 | AYA73395 | *"Mycoaspiviridae"* |
| Rhizoctonia solani  negative-stranded virus 1 | ALD89129.2 | *"Mycoaspiviridae"* |
| Plasmopara viticola lesion  associated mycoophiovirus 5 | MN585281 | *"Mycoaspiviridae"* |
| Colletotrichum associated  Negative-stranded RNA virus 2 | UYD21357 | *"Mycoaspiviridae"* |

**Table S2** List of PCR primers used for viral contig detection

| **Contig number** | **Primer Forward (5’-3’)** | **Primer Reverse (5’-3’)** | **Product Size (bp)** |
| --- | --- | --- | --- |
| Contig48674 | GTGAAGATGGTTGCCGAA | GTGTTCTATGTAATCGCTCGCT | 965 |
| Contig8039 | CACAGCACTTACACGCAAA | GCACTTCATTACAGCGGAT | 829 |
| Contig18916 | CCTTCAGGAGTTCATCACG | GCGAGTTGCTCTCAGTTGTT | 873 |
| Contig4961 | CCAACACCGCTTTCAGAAC | CCAGTGATGGACAGGATTACC | 859 |
| Contig14885 | CCATTTCACAGGACGACA | GAAGCCATAACTCTCAAGCG | 988 |
| Contig13902 | GAGACGGAGGAGATGACAA | CCGAGAGGAAGGAAAGAGA | 878 |
| Contig3839 | CGGAATGCTCAAGAATCTCT | TTTCACGAACCTTACCTGGT | 728 |
| Contig1940 | TTACAGGTCTCGCTTGGAG | CGTCAACGACTGCTTCAA | 765 |
| Contig1392 | ACGGACTATTCCTCCAACG | CGGGTTCTCTGACTATGGTC | 792 |
| Contig33103 | GCTTATCCGTCACCTCGTAG | GCTGTATTCACATTGTCTCACC | 1033 |
| Contig23320 | CTTACCGTGGATGAAATCACT | TCAGACAGCACTTAGACCTTT | 876 |
| Contig28389 | AGCGATTCGGCTTCACAGT | GCAGAGCGTAGTTGTTCCACT | 994 |
| Contig989 | TGGGTATCGCAGTAATCGTC | CCATCATCCTTTCAATCGTG | 913 |
| Contig534 | CTCCATCTAAACTGCCTTCTG | CTTACGGCTCCTACTTTCGT | 759 |
| Contig57982 | ACCAAATGCTAAGGAAGCC | AGACCACATCGCCAGAAT | 525 |
| Contig3871 | CATCAACTCACTCATTACGACC | CGATACGGACTTGGCAAA | 523 |
| Contig334 | ATTCAGAGTCAGACCGAGAGA | TGGCATCTACTTTGTCCCA | 1223 |
| Contig1200 | CTGCTGACCTTGAACACCT | AGGATTTATGGGCTTCTGG | 972 |
| Contig5716 | AAGTCGGCATCCTCTTCA | TGGCGTCAAGTAACATCG | 885 |
| Contig4493 | AGATGGCAGCGTAGCGAAT | ACGGTGAACAACCCTCTTCC | 833 |
| Contig4539 | AGTGGGTGAGTAAGGAGTGC | GAGGTTGAACATTCGCCTA | 743 |
| Contig1494 | GCCAGTTTCTGATTTCCCTA | TTTCACCTTCGGTAGCGT | 823 |
| Contig1895 | CTATTCACCATCTAACCTGGGT | TTTCTAACTCATCCGTCGC | 795 |
| First_Contig593 | CCCACCATTGATACCAGTTAC | TGGATTGTCATTGAGGTCTG | 755 |
| Contig1205 | TCCAGAACACGAAGAGCAA | TCCGAACCACAGGGTATGT | 994 |
| Contig70151 | CCGTCTCCACATAAGTCGT | TGTTTGGGATGACACTCG | 1009 |
| Contig43866 | AAGAATGCGGGTGCCTTT | AGAGCGTGTTGCGTGTTGT | 879 |
| Contig1235 | CAGAAGAATGTCAAACCCACT | CGAAGTCATCAATGTGCGTAG | 855 |
| Contig4308 | ACTGACTCTGTAGCACCGCT | TGACACTCACCCAATACTATCG | 1136 |
| Contig609 | CACCTACAGTCATTATCAAGG | TAACAGAAGTAACAGGGCTCC | 990 |
| Contig3546 | ATCCTCACAAAGGGCAAG | GCCTCACTCTCCCATAAGAC | 787 |
| Contig6707 | GGGACTCTTTGCTGCTACCT | GGTCTGTTCAGGAAATAAGGG | 983 |
| Contig2482 | TGGTGTTGTTGGAGCACA | AAGTCCTTGTCCTCGCAGT | 1857 |
| Contig16587 | AATCTCAACTTCCTATGCCG | TCGTCTGAATGTGCCATC | 923 |
| Contig8685 | CGGAAAGTCTATTGTCTCAGG | CAAGATGGAAGAAGAGGCAC | 1251 |

**Table S3** Viruses used for pairwise identity comparisons

| **Virus Name** | **Abbreviation** | **Accession number** |
| --- | --- | --- |
| Saccharomyces 23S RNA narnavirus | ScNV23S | NP_660177 |
| Saccharomyces 20S RNA narnavirus | ScNV20S | NP_660178 |
| Rhizopus microsporus 23S narnavirus | RmNV23S | QBC65281 |
| Rhizopus microsporus 20S narnavirus | RmNV20S | QBC65280 |
| Erysiphe necator associated narnavirus 37 | EnNV37 | QJT93769 |
| Erysiphe necator associated narnavirus 42 | EnNV42 | QJT93774 |
| Erysiphe necator associated narnavirus 49 | EnNV49 | QJT93781 |
| Plasmopara viticola lesion associated narnavirus 7 | PvaNV7 | QIR30286 |
| Aspergillus flavus narnavirus 1 | AfNV1 | UAW09567 |
| Aspergillus creber narnavirus 1 | AcNV1 | BDB16246 |
| Rhizopus oryzae narnavirus 1 | RoNV1 | BDF97660 |
| Fusarium poae narnavirus 1 | FpNV1 | YP_009272902 |
| Alternaria tenuissima narnavirus 1 | AtNV1 | WAK75255 |
| Zhejiang mosquito virus 3 | ZjMV3 | YP_009333331 |
| Mitovirus sp. | unamed mitovirus | MN034983 |
| Entomophthora muscae mitovirus 5 | EnmuMV5 | BK010733 |
| Entomophthora muscae mitovirus 2 | EnmuMV2 | BK010730 |
| Sclerotinia sclerotiorum mitovirus 39 | SsMV39 | MT646411 |
| Neofusicoccum parvum mitovirus 3 | NpMV3 | MW175882 |
| Nigrospora oryzae mitovirus 2 | NoMV2 | MH823902 |
| Fusarium mangiferae mitovirus 3 | FmMV3 | MZ493903 |
| Fusarium sambucinum mitovirus 5 | FsamMV5 | LC596829 |
| Ophiostoma mitovirus 4 | OMV4 | AJ132754 |
| Alternaria arborescens mitovirus 1 | AaMV1 | LC145036 |
| Cryphonectria parasitica mitovirus 1 | CpMV1 | L31849 |
| Botrytis cinerea mitovirus 1 | BcMV1 | EF580100 |
| Ceratobasidium mitovirus A | CbMV-A | KU291923 |
| Geopora sumneriana mitovirus 1 | GsMV1 | MN043682 |
| Ophiostoma mitovirus 7 | OMV7 | KF031943 |
| Ditton virus | DiV | AWA82278 |
| Mucor phasmavirus A | MuPVA | QED42998 |
| Guyuan tick virus 1 | GyTV1 | UYL95512 |
| Phasmaviridae sp. | unamed Phasmaviridae sp. | WAK75648 |
| Botrytis cinerea  negative-stranded RNA virus 6 | BcNSRV6 | QJT73694 |
| Botrytis cinerea  negative-stranded RNA virus 9 | BcNSRV9 | MT157409 |
| Botrytis cinerea bocivirus 1 | BcBV1 | QJT73693 |
| Phytophthora condilina  negative-stranded RNA virus 9 | PcNSRV9 | QTT61001 |
| Erysiphe necator associated  negative-stranded RNA virus 24 | EnaNSRV24 | QJW70356 |
| Penicillium roseopurpureum  negative ssRNA virus 1 | PrNSRV1 | AYP71800 |
| Rhizoctonia solani  bunya/phlebo-like virus 1 | RsBPlV1 | MK507779 |
| Fusarium graminearum  negative-stranded RNA virus 1 | FgNSRV1 | MF276904 |
| Sclerotinia sclerotiorum  negative-stranded RNA virus 1 | SsNSRV1 | NC_025383 |
| Soybean leaf-associated  negative-stranded RNA virus 1 | SlaNSRV1 | KT598225 |
| Hymenopteran phasma-related virus | HpPrV | MW274746 |
| Coniothyrium diplodiella  negative-stranded RNA virus 1 | CdNSRV1 | QDB75015 |
| Tulasnella bunyavirales-like 1 | Tbl1 | QPB44676 |

**Table S4** Viruses used for multiple alignments

| **Virus Name** | **Abbreviation** | **Accession number** |
| --- | --- | --- |
| Saccharomyces 23S RNA narnavirus | ScNV-23S | NP_660177 |
| Saccharomyces 20S RNA narnavirus | ScNV-20S | NP_660178 |
| Blechmonas luni narnavirus 1 | BlNV1 | MG967337 |
| Aspergillus flavus narnavirus 1 | AfNV1 | UAW09566/UAW09567 |
| Oidiodendron maius splipalmivirus 1 | OmSPV1 | QNN89179/QNN89180 |
| Botrytis cinerea binarnavirus 5 | BcBNV5 | QJT73728/QTP72365 |
| Cryphonectria cubensis mitovirus 2a | CcMV2a | AAR01973 |
| Ophiostoma mitovirus 3a | OMV3a | NP_660176 |
| Colletotrichum gloeosporioides  ourmia-like virus 1 | CgOLV1 | QDW80875 |
| Botrytis ourmia-like virus | BOLV | QLF49182 |
| Phoma matteucciicola ourmia-like virus 1 | PmOLV1 | QIP68359 |
| Magnaporthe oryzae ourmia-like virus | MOLV | YP_009667033 |
| Pestalotiopsis botourmiavirus 2 | PBV2 | QTH80196 |
| Alternaria alternata hypovirus 1 | AaHV1 | QFR36339 |
| Fusarium sacchari hypovirus 1 | FsHV1 | QIQ28422 |
| Fusarium graminearum hypovirus 1 | FgHV1 | AGC75065 |
| Bipolaris oryzae hypovirus 1 | BoHV1 | QDY81493 |
| Cryphonectria hypovirus 1 | CHV-1 | NP_041091 |
| Sclerotinia sclerotiorum endornavirus 1 | SsEV1 | YP_008169851 |
| Oryza sativa endornavirus | OsEV | D32136 |
| Rhizoctonia solani endornavirus 2 | RsEV2 | KT823701 |
| Chalara elegans endornavirus 1 | CeEV1 | ADN43901 |
| Sclerotinia sclerotiorum deltaflexivirus 1 | SsDFV1 | NC_038977 |
| Fusarium graminearum deltaflexivirus 1 | FgDFV1 | NC_030654 |
| Lentinula edodes deltaflexivirus 1 | LeDFV1 | MN744723 |
| Pleosporales megabirnavirus 1 | PMBV1 | ALO50147 |
| Sclerotinia sclerotiorum megabirnavirus 1 | SsMBV1 | YP_009143529 |
| Rosellinia necatrix megabirnavirus 2 | RnMBV2 | LC062704 |
| Alternaria alternata virus 1 | AaV1 | YP001976142 |
| Fusarium poae alternavirus 1 | FpAV1 | YP009272952 |
| Fusarium graminearum alternavirus 1 | FgAV1 | YP009449439 |
| Fusarium graminearum  negative-stranded RNA virus 1 | FgNSRV1 | MF276904 |
| Sclerotinia sclerotiorum  negative-stranded RNA virus 1 | SsNSRV1 | NC_025383 |
| Soybean leaf-associated  negative-stranded RNA virus 1 | SlaNSRV1 | KT598225 |
| Citrus concave gum-associated virus | CCGaV | AYN78565 |
| Citrus virus A | CiVA | QEU56214 |
| Watermelon crinkle leaf-associated virus 1 | WCLaV-1 | ASY01340 |
| Rhizoctonia solani bunya/phlebo-like virus 1 | RsBPLV1 | MK507779 |
| Coniothyrium diplodiella  negative-stranded RNA virus 1 | CdNSRV1 | QDB75015 |
| Botrytis cinerea  negative-stranded RNA virus 9 | BsNSRV9 | MT157409 |
| Penicillium roseopurpureum  negative ssRNA virus 1 | PrNSRV1 | AYP71800 |
| Tomato spotted wilt orthotospovirus | TSWV | NP_049362 |
| Rhizoctonia solani negative-stranded virus 1 | RsNSV1 | ALD89129.2 |
| Colletotrichum associated  negative-stranded RNA virus 2 | CaNSRV-2 | UYD21357 |
| Fusarium poae negative-stranded virus 1 | FpNSV1 | YP009272911 |
